# A Copper‐Complexing Lipid Unmasks the Ethanol Hidden Within Liposomes and Highlights the Relevance of Fabrication Method on the Performance of Lipophilic Functional Molecules

**DOI:** 10.1002/smsc.70357

**Published:** 2026-08-04

**Authors:** Leonardo Severini, Beatrice Simonis, Davide Lanera, Marco Campanile, Pompea Del Vecchio, Federica Di Mascolo, Isabella Ossani, Francesca D’Acunzo, Cecilia Bombelli, Simona Sennato

**Affiliations:** ^1^ Institute for Complex Systems (ISC) National Research Council (CNR) c/o Department of Physics Sapienza University of Rome Rome Italy; ^2^ Institute for Biological Systems (ISB) National Research Council (CNR) Secondary Office of Rome‐Reaction Mechanisms c/o Department of Chemistry Sapienza University of Rome Rome Italy; ^3^ Department of Chemical Science and Technologies University of Rome “Tor Vergata” Rome Italy; ^4^ Department of Chemical Sciences University of Naples Federico II Naples Italy

**Keywords:** bilayer properties, liposomes, metal ion binding, microfluidics, thin film hydration

## Abstract

Liposomes are traditionally produced batch‐wise using laborious bulk methods. In contrast, microfluidics (*μ*F) enables operator‐independent, continuous, and scalable production of liposomes with narrow size distributions, aided by advanced chip technology. However, *μ*F fabrication may leave traces of the water‐miscible organic solvent used to dissolve lipids. Thereby, benign solvents such as ethanol are employed, and post‐processing ensures acceptable limits of residues. Nonetheless, residual ethanol can influence liposome structure, stability, and the performance of component functional molecules. These effects are typically investigated by microscopy, scattering, and diffraction techniques, not suited for high‐throughput applications. Here, the impact of liposome fabrication method on the incorporation of 2‐(hydroxyimino)‐3‐octyltridecanal (HIOTD) and on its performance as a Cu(II) chelator is assessed using UV–vis spectrometry. DMPC liposomes and a commercial *μ*F micromixer chip are employed as a representative case, with solvent‐free thin film hydration (TF) as a benchmark for comparison. Combined DLS, calorimetry, and fluorescence anisotropy analyses reveal physicochemical differences between *μ*F and TF liposomes, while UV–vis spectroscopy highlights variations in incorporation and metal‐binding performance of HIOTD. These findings demonstrate that the liposome fabrication route critically affects the functional behavior of embedded molecules and propose a convenient UV–vis approach for comparative evaluation of fabrication methods, in view of their clinical translation.

## Graphical abstract

Liposomes of the same composition obtained with microfluidics (μF) and thin film hydration methods exhibit different physicochemical properties, due to residual traces of ethanol from the μF protocol. This affects the insertion of a synthetic lipophilic ligand (HIOTD) and its ability to complex Cu(II). Liposome fabrication techniques should be carefully evaluated to fully meet the requirements of specific applications.

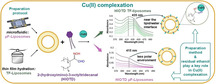

## 1 Introduction

Liposomes are nanosized spherical vesicular structures, composed of one or more lipid bilayers, enclosing a fraction of the surrounding aqueous medium [1, 2]. They are among the most effective and multipurpose drug delivery systems thanks to their biocompatibility, to the modulation of lipid composition, and to the ability to protect both hydrophilic and lipophilic drugs, which can be either incorporated within the liposome or conjugated to the surface of the vesicle. Liposomes cover mostly intravenous and intramuscular delivery routes along with ever‐evolving novel nanomaterials [3], recently comprising also injectable nanogels [4]. Since the earliest investigations, it has been well known that composition affects many physicochemical properties of liposomes, but also that the production method has a deep impact on the final size, polydispersity, lamellarity, and stability of the vesicles formed [1]. Often overlooked, this aspect is not merely a technical detail but a crucial factor in selecting the most suitable protocol. Furthermore, fine control over manufacturing parameters is essential to ensure a final product with satisfactory characteristics.

Over the past few decades, various techniques have been developed for the preparation of liposomes [1], such as thin‐film hydration, detergent removal, solvent injection and reverse‐phase evaporation. The oldest, most common, and simplest way to prepare liposomes is the thin‐film (TF) hydration method (the so‐called Bangham method), which produces homogeneous vesicles with controlled sizes. However, liposome production through the TF method often exhibits significant limitations in terms of process reproducibility and scalability. In TF method, all the relevant steps of the process occur in bulk, and achieving ideal mixing conditions may be challenging due to a nonuniform distribution of reagents, the presence of temperature gradients, and variable shear forces [5, 6]. These inhomogeneities may result in significant batch‐to‐batch variability. Moreover, the whole process involves several manual steps to yield populations of appropriate size, and nevertheless the degree of size selection is limited [5].

To overcome the limitations described above, microfluidics (*μ*F) has emerged as a promising alternative method [7]. In this case, the hydrodynamically driven solvent exchange between the solution of lipids in a water‐miscible organic solvent and an aqueous buffer enables the formation of liposomes with controlled mean sizes, narrow size distributions, and well‐defined lamellarity, in a single step [8, 11]. Unlike bulk processes, *μ*F allows for continuous production under highly reproducible conditions, minimizing batch‐to‐batch variability [12]. The possibility to finely tune parameters such as flow rate ratio (FRR), defined as the ratio of the aqueous‐to‐organic flow rate, as well as the chip architecture, offers an unprecedented level of control over final liposome characteristics, compared to bulk counterparts [8, 10, 13, 15]. Controlling liposome size is crucial in drug delivery, as it directly affects their uptake by cells, transport within the body, susceptibility to immune system‐mediated clearance, and accumulation at target sites [8, 11, 16, 17].

*μ*F is also particularly promising for obtaining liposomes in sterile conditions. In principle, the closed and miniaturized microfluidic devices inherently reduce the risk of contamination, making them especially suitable for applications requiring aseptic processing, such as pharmaceutical and biomedical formulations. After the production of liposomes, sterilization can be achieved by integrating in‐line sterile filtration as part of the on‐chip process [18], thus avoiding conventional sterilization techniques that may compromise the physicochemical and biopharmaceutical properties of liposomes [19, 20]. A further advantage of *μ*F is its complete automation, which allows scale‐up through parallelization, an aspect which is overlooked at the laboratory scale, but critical for potential industrial translation of this technology [21, 22]. Key issues associated with *μ*F are low final liposome concentration and limitations in the choice of lipids, which must be soluble in water‐miscible organic solvents of low toxicity, such as alcohols [16]. Moreover, elution in alcohol/water mixtures can make it necessary to implement post‐processing steps aimed at removing alcohol, since its presence can affect the stability of liposomes, their physicochemical characteristics, and may interfere with downstream applications [14]. Dialysis, dilution, and tangential flow filtration may still leave traces of organic solvent in the liposome bilayer. For this reason, the selection of organic solvents for lipid dissolution has emerged as a key determinant of liposome properties during microfluidic production. In recent works, the systematic evaluation of different alcohols, i.e., methanol, ethanol, isopropanol, and their binary mixtures, revealed that decreasing solvent polarity leads to increased average particle size [23]. Moreover, the so‐called lipid “interdigitation” phenomenon occurring in membranes formed by saturated phospholipids is promoted by short alcohol chain lengths and leads to highly unstable vesicles [24, 26]. Despite this evidence, only a few studies investigate the role of residual alcohol traces in affecting the integrity of liposomes produced by *μ*F and their stability over time [24, 26, 27]. On the other hand, drug and multidrug encapsulation of liposomes obtained with *μ*F techniques are being increasingly investigated. The focus of the current literature is typically on encapsulation efficiency, stability, and in vitro and in vivo performance of the formulations [14], whereas the impact of the *μ*F preparation method on the properties of the lipid assemblies at the nanometric level is often overlooked. Extremely detailed information on the internal organization of lipid bilayers and on the positioning of functional molecules within is gained through powerful techniques, such as cryo‐TEM, X‐ray diffraction, neutron scattering, and solid‐state NMR [28, 29]. However, these techniques are costly, time‐consuming, and may impose restrictions in terms of concentration and other experimental parameters. Furthermore, they require a strong effort in the interpretation of results in order to handle potential artifacts and to model the data correctly. Notwithstanding their indisputable value in detailed structural studies, they can hardly be included in a high‐throughput lab routine aimed at optimizing the inclusion and performance of functional molecules in lipid vesicles. Thereby, the aim of this work is to pinpoint critical issues in the transition from the well‐established TF method (the gold standard in our Lab, so far) to less cumbersome *μ*F liposome production. The following points are discussed: (i) ethanol removal after *μ*F fabrication; (ii) the effect of fabrication method (TF vs*.* *μ*F) on fluidity of the lipid bilayer and liposome stability; (iii) highlighting the relevance of fabrication method on the performance of a lipophilic functional molecule, acting as a probe for routine UV–vis spectrometry.

To this end, vesicles were formulated with 1,2‐dimyristoyl‐*sn*‐glycero‐3‐phosphocholine (DMPC) lipid, commonly used in many approved liposomal drugs, and cholesterol as an excipient for tuning bilayer fluidity [30, 31]. As for the *μ*F device, we used one of the most popular commercial micromixers for our case study. To better understand the effect of the residual ethanol on liposomes produced by *μ*F, the outcomes of TF and *μ*F preparation methods were directly compared. Unlike *μ*F‐liposomes, those manufactured by the TF hydration method are alcohol‐free and serve as a control, since organic solvents are completely removed under vacuum prior to vesicle formation. Focusing on liposomes produced by *μ*F, the physicochemical properties of the vesicles and the order/fluidity of the lipid bilayer were evaluated immediately after preparation and after a dialysis step performed to remove the alcohol. Our main concern is the effect of residual ethanol, most often used in the literature, on the properties of *μ*F‐liposomes [32].

**FIGURE 1:**
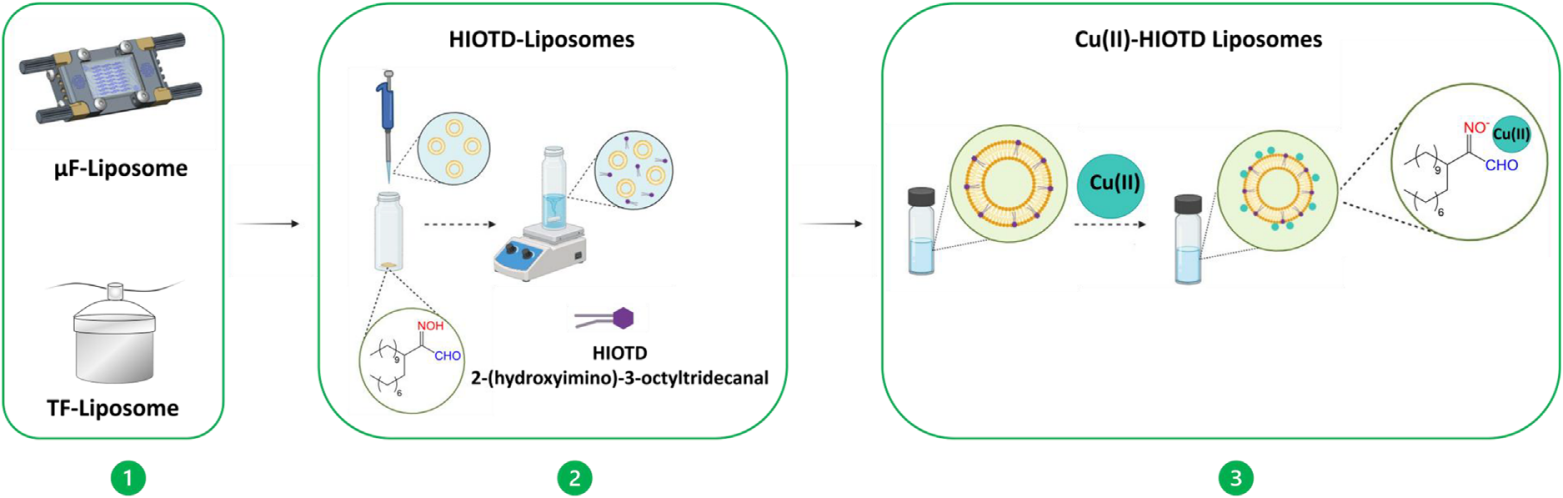
Schematic illustration of the three‐step procedure used to obtain liposomes functionalized for Cu(II) complexation. **1)** Liposome preparation by two different techniques: microfluidics (μF), using a Dolomite microfluidic platform and a micromixer chip, and thin‐film hydration followed by extrusion (TF). **2)** Functionalization of preformed liposomes by incubation with the metal‐complexing lipid HIOTD. **3)** Addition of Cu(II) to obtain Cu(II) HIOTD‐ liposomes. The evaluation of metal‐complexation capability of liposomes was performed through Cu(II) binding experiments.

As a further step, the in‐house metal‐complexing lipid 2‐(hydroxyimino)‐3‐octyltridecanal [33], (Figure 1, HIOTD) was incorporated in liposomes to enable additional surface activity and probe its dependence on the liposome preparation method (TF or *μ*F). The structure of HIOTD is quite simple, as its alkyl portion resembles that of the hydrophobic chains of DMPC, thus minimizing the perturbation of the lipid bilayer upon its incorporation through post‐processing incubation. It has to be noted that this loading method allows the direct comparative study of internal and surface properties of TF‐ or *μ*F‐nanostructures. Besides being a tool for physicochemical investigation of liposomes, the incorporation of HIOTD in the lipid bilayer may prove relevant for the realization of novel theranostic systems, with potential applications both in therapy and diagnostics, depending on the nature of the complexed metal. As a case study, we focus on Cu(II) complexation [34], thus taking advantage of the convenient spectral properties of copper complexes and the availability of experimental data on other systems endowed with the 2‐(hydroxyimino)aldehyde (HIA) functional group [35, 36]. As an added value, Cu(II)‐complexing liposomes are of considerable interest in anticancer therapy based on reactive oxygen species (ROS) production [37, 38], as antimicrobial nanomaterials [39, 40], and as nuclear imaging probes with ^64^Cu [41, 43]. In fact, as demonstrated in previous studies, the 2‐(hydroxyimino)aldehyde (HIA) functional group is able to bind Cu(II) in medium‐ to low‐polarity environments, such as acetonitrile, as well as when anchored to the repeat units of the hydrophobic block of amphiphilic copolymers [33, 35, 36]. This coordination is evidenced by the presence of a sharp and intense LMCT band in the 450–400 nm region of the UV–vis spectrum.

## 2 Results and Discussion

### 2.1 Influence of Production Method on Liposomes Stability

Liposomes were prepared with DMPC and DMPC:chol, for tuning bilayer fluidity [30, 31], by considering two different procedures, µF and thin‐film hydration followed by extrusion (TF) (Table 1) to investigate the influence of the preparation method on the physicochemical properties of liposomes. To understand the role played by the residual ethanol, inevitably retained in microfluidic formulations, µF‐liposomes were studied before and after the dialysis step for ethanol removal. Throughout the investigation, µF‐liposomes have been compared with those prepared by thin film hydration (TF‐liposomes), where ethanol is absent. Indeed, the TF method involves solvent evaporation to form a lipid film, followed by hydration with an aqueous solvent phosphate buffer (PB solution 0.01 M, pH 7.4), and extrusion through a 100 nm membrane. In contrast, *μ*F exploits a rapid and controlled mixing of lipid‐ethanol (the organic phase) and PB solution (0.01 M, pH 7.4) (aqueous phase) in a glass chip, at room temperature, resulting in an ethanol content of 8% v/v in the final dispersing media, based on the employed FRR. With both methods, the final concentration of liposomes is 1 or 2 mM.

**TABLE 1:** Physicochemical properties of TF‐ and µF‐liposomes, produced according to the indicated protocol. Average size, PDI and ζ‐potential are presented as mean ± SD (*n* = 3). Errors on Δ*H*_m_ and *T*_m_ were always below 10%.

Sample mole ratio	Protocol	***D***_**h**_ **, ** **nm**	PDI	ζ‐potential, mV	**Δ*H***_**m**_ **, ** **kJ/mol**	***T***_**m**_ **, ** **°C**
TF‐DMPC	TF	111 ± 1	0.12 ± 0.01	−7.3 ± 0.7	14.1	24.4
TF‐ DMPC:chol (9.5:0.5)	TF	117 ± 1	0.09 ± 0.00	−6 ± 1	16.3	22.4
µF‐DMPC	µF	119 ± 1	0.06 ± 0.02	0.4 ± 0.1	125	23.9
µF‐DMPC:chol (9.5:0.5)	µF	102 ± 1	0.09 ± 0.01	0.3 ± 0.1	110	22.8
µF‐DMPC	µF + dialysis	121 ± 1	0.06 ± 0.01	0.3 ± 0.1	97	22.5
µF‐DMPC:chol (9.5:0.5)	µF + dialysis	104 ± 1	0.08 ± 0.01	0.5 ± 0.1	46.9	21.7

The hydrodynamic diameter (*D*_h_), polydispersity index (PDI), and *ζ*‐potential of liposomes were monitored by dynamic and dielectrophoretic light scattering measurements (DLS, DELS) to determine their stability or tendency to aggregate over 20 days. This is a fundamental analysis for evaluating the effectiveness of a preparation method to produce liposomes as a drug carrier. All samples were stored at 20 °C, slightly below the melting temperature of DMPC (*T*_m_ = 24 °C) [44]. Indeed, in these storage conditions, lipids were in an ordered gel phase and possible destabilizing effects triggered by residual alcohol molecules could be highlighted [24].

**FIGURE 2:**
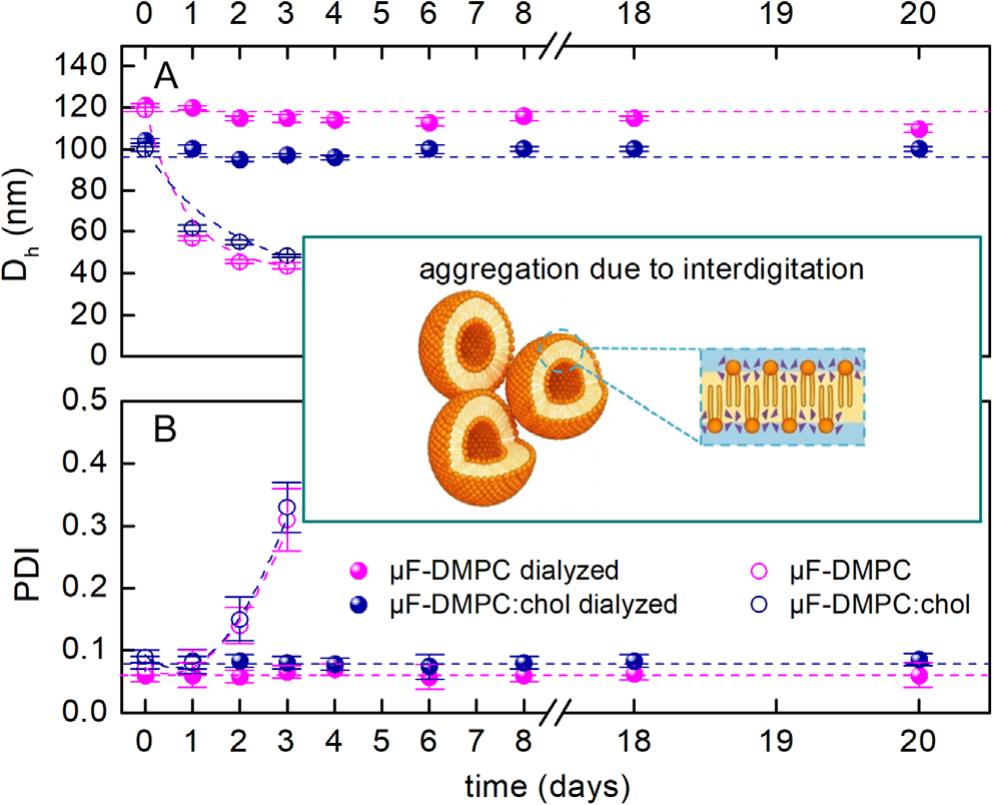
(A) Hydrodynamic diameter (*D*_h_) and (B) polydispersity index (PDI) of µF‐liposomes, before and after dialysis. After 4 days, undialyzed µF‐liposomes undergo aggregation due to interdigitation, as shown by the schematic illustration of the phenomenon (generated using Gemini, Google AI) with violet triangles indicating ethanol molecules. Error bars are the standard deviations of three repeated measurements. If not visible, the error bars are smaller than the symbols used in the graph.

Soon after the preparation, DMPC and DMPC:chol µF‐liposomes showed a mean hydrodynamic diameter in the range 100–120 nm, comparable to that of TF‐liposomes, and a narrow size distribution (PDI < 0.1), similarly to the one of TF‐liposomes, while the *ζ*‐potential was different, being close to 0 mV or slightly negative (≅−7 mV), for µF‐ and TF‐liposomes, respectively (Table 1). In the subsequent 3 days, the two undialyzed µF‐formulations showed similar behaviors, with *D*_h_ rapidly decreasing (Figure 2A), then increasing up to several microns on longer times, and a PDI exceeding 0.3. Size distribution analysis performed by the NNLS algorithm on these samples at the onset of the aggregation regime confirms the presence of micrometric objects (Figure S1). In these samples, it can be hypothesized that the presence of ethanol molecules, randomly distributed within the bilayer, may act as a driving force for the formation of local inhomogeneities and curvature fluctuations, which promote membrane fragmentation and subsequent generation of smaller vesicles. At this stage, subsequent interdigitation involving the ethanol molecules retained within the bilayer may occur. Indeed, the presence of short‐chain alcohols such as ethanol strongly promotes membrane interdigitation in saturated phospholipids such as DMPC, thus affecting the shelf‐life of liposomes [24, 45]. Smith et al. demonstrated that vesicles can be fully converted into an interdigitated structure when the ethanol threshold concentration is about 6.6% v/v [46], very close to the conditions used here (8% v/v). DMPC with its saturated chains was therefore likely to produce interdigitated structures, where acyl chains extend beyond the bilayer midplane and interpenetrate into the opposing monolayer, thus leading to aggregation of liposomes within 3 days after preparation [46, 47]. Furthermore, due to the neutral charge of our liposomal formulations, electrostatic interactions can be excluded as a contributing factor to stability. This phenomenon occurs only with µF‐liposomes, and it is insensitive to the presence of cholesterol in the bilayer. Conversely, the physicochemical properties of TF‐liposomes, reported in Table 1, remained almost unchanged for at least 20 days for both DMPC and DMPC:chol, as expected.

To better highlight the influence of ethanol on the organization of lipids inside the liposome bilayer, alcohol was removed just after *μ*F preparation through a dialysis step above the melting temperature of DMPC (*T*_m_ = 24 °C), so that the release of ethanol trapped within the bilayer would be facilitated by the liquid–crystalline phase. No variation of *ζ*‐potential is observed before and after dialysis, probably related to the absence of reorientation of PC dipole and variation of surface charge (see Table 1). As shown in Figure 2, the size and the PDI of the vesicles after dialysis remain almost unchanged, supporting the efficacy of the purification method. The stability of dialyzed µF‐liposomes significantly increases up to at least 20 days, becoming comparable with that of TF‐liposomes. This finding provides indirect evidence for the removal of ethanol from the liposome suspending medium, in line with results reported for other liposomal vesicles [48]. The effectiveness of the dialysis procedure in removing ethanol from the liposome suspensions was assessed by comparing the conductivity of dialyzed µF‐ and TF‐liposomes (Table S1). For suspensions of neutral liposomes, conductivity is primarily determined by the composition of the dispersing medium. Prior to purification, µF‐liposomes exhibited lower conductivity than TF‐liposomes, consistent with the presence of ethanol in the medium, which reduces the conductivity of the bulk solution. After dialysis, no significant differences were observed. These results demonstrate that dialysis efficiently removed ethanol from the dispersing medium of µF‐liposome suspension.

While conductivity measurements point to a thorough removal of ethanol from the aqueous bulk, traces of ethanol may still be trapped within the lipid bilayer. Fluorescence anisotropy measurements were employed to indirectly monitor the efficiency of ethanol removal by dialysis from the bilayer and obtain information on the arrangement of lipid aliphatic chains within the bilayer. To explore the fluidity of the inner hydrophobic region of the lipid bilayer [49, 51], the (DPH) probe was used [52], whose value reaches the maximum anisotropy value of 0.4 in rigid systems and decreases when DPH is distributed in more disordered environments [53].

**FIGURE 3:**
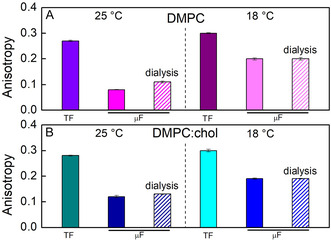
Fluorescence anisotropy for DPH probe in (A) TF‐DMPC and µF‐DMPC liposomes and (B) TF‐DMPC:chol and µF‐DMPC:chol liposomes, dialyzed or not (patterned and full bars, respectively), measured at two different temperatures above and below the melting temperature of DMPC.

Figure 3 shows the results of DPH anisotropy determined at two different temperatures, 25 and 18 °C, above and below the melting temperature of DMPC, respectively. Results collected at *T* = 25 °C indicate that TF‐liposomes exhibit anisotropy values higher than those of µF‐liposomes, as expected, because ethanol, inevitably present in the bulk solution of *μ*F preparation, can penetrate inside the bilayer, affecting the organization and packing of the lipids and increasing their fluidity. After dialysis at *T* = 25 °C, the anisotropy value of the µF‐liposomes increases, but remains lower than that of TF‐liposomes, thus indicating partial removal of ethanol in the bilayer. As shown in Figure 3, when dialysis is carried out at *T* = 18 °C, the anisotropy values for µF‐liposomes determined at *T* = 18 °C are significantly higher than those recorded at *T* = 25 °C. Interestingly, the relative increase of anisotropy values is larger for µF‐liposomes, irrespective of dialysis, thus suggesting that ethanol acts as a stiffening enhancer. In fact, at 18 °C no difference is observed before and after dialysis, testifying to the ability of the bilayer to pack in a more rigid and compact structure also in the presence of small amounts of ethanol, due to the strong attractive interaction between lipid aliphatic chains. To sum up, above the melting temperature of DMPC, alcohol strongly promotes disorder in the inner bilayer region; below this threshold, instead, it stiffens the lipid tails. Overall, these results suggest that the hydroxyl group of ethanol is oriented at the bilayer interface, while its short aliphatic chain intercalates between the lipid acyl chains, making *μ*F‐liposomes more flexible than TF‐liposomes. These findings are consistent with the literature, which highlights the fluidizing role of alcohol in ethosomes, where it is present in higher concentration up to 45%, to enhance vesicle deformability and flexibility, thereby improving their ability to penetrate the skin [54].

Last, the membrane stiffening effect of cholesterol is observed only at *T* = 25 °C in µF‐liposomes, where minimal differences in fluorescence anisotropy values between the two formulations (DMPC and DMPC:chol) are observed.

Further information about the role of ethanol and its partitioning into lipid bilayers was sought by differential scanning calorimetry (DSC) (see Figure S2 for DSC thermograms). The obtained parameters are reported in Table 1. For TF‐DMPC, a lamellar‐to‐ripple gel pretransition was detected at 13.6 °C with an enthalpy change of 0.6 kJ/mol, followed by the gel‐to‐liquid crystalline main transition centered at 24.4 °C and with an enthalpy change of 14.8 kJ/mol. Although these values differ from the commonly reported values for DMPC multilamellar vesicles, they are consistent with differences in vesicle architecture (e.g., lamellarity/curvature and associated heterogeneity), which are known to affect both transition temperature and enthalpy in extruded systems [55]. The lamellar‐to‐ripple pretransition was not resolved in any of the other lipid formulations, indicating that changes in composition (ethanol and/or cholesterol) and vesicle structure are sufficient to perturb this transition.

Nondialyzed µF‐DMPC are characterized by an apparent main transition occurring at a lower *T*_m_ (23.9 °C) and with a considerably higher enthalpy change (125 kJ/mol). This behavior is consistent with the presence of 8% v/v ethanol in the suspending media, above the reported threshold for the alcohol‐induced perturbation of DMPC phase behavior and the promotion of bilayer interdigitation [56]. Under these conditions, overlapping contributions from ripple/gel and interdigitated states could deeply affect the observed enthalpy changes relative to the single main transition. This could also be attributed to a redistribution of the ethanol molecules in the bilayer passing from the gel to liquid phases. Consistently, DPH anisotropy indicates that core rigidity varies more strongly with temperature in µF‐liposomes than in TF‐liposomes. After ethanol removal by dialysis, *T*_m_ decreases to 22.5 °C, and the apparent enthalpy change decreases to 97 kJ/mol, suggesting that residual ethanol still perturbs the gel phase, although to a reduced extent. Cholesterol‐containing systems show the same overall trend, with two notable differences: (i) dialyzed µF‐liposomes exhibit a *T*_m_ slightly lower than the corresponding TF samples; (ii) dialysis produces a larger reduction in the enthalpy change (to 46.9 kJ/mol), likely due to the attenuation of ethanol‐driven perturbations in the presence of cholesterol [57].

All these findings highlight the pivotal role of residual ethanol in determining the properties of DMPC‐based liposomes prepared by *μ*F. Although µF‐ and TF‐liposomes show comparable initial sizes, ethanol retained in µF‐liposomes induces bilayer interdigitation and instability, leading to fragmentation and size heterogeneity after a few days. Fluorescence anisotropy and DSC analyses performed on as‐prepared µF‐liposomes demonstrate that ethanol disturbs lipid packing in µF‐ liposomes, increasing bilayer fluidity above *T*_m_ and promoting the formation of rigid interdigitated phases below *T*_m_. Dialysis above the lipid melting temperature removes ethanol in the bulk aqueous phase, but only partially reduces its content in the bilayer. Indeed, *μ*F‐liposomes remain more flexible than TF‐liposomes, indicating that a fraction of ethanol remains trapped within the bilayer even after dialysis.

### 2.2 Incorporation of HIOTD in Liposomes

HIOTD was incubated with TF‐liposomes or dialyzed µF‐liposomes obtained with DMPC and DMPC:chol (Figure 1, step 2). Table 2 reports the data on size, PDI, and *ζ*‐pot of the four different samples, and their variation (Δ) induced by the incorporation of the HIOTD molecule.

**TABLE 2:** Name, composition and values of hydrodynamic diameter (*D*_h_), polydispersity (PDI) and ζ‐potential (ζ‐pot) of HIOTD‐liposomes and their variation Δ with respect to the values of liposomes measured in the absence of HIOTD.

Sample molar ratio	*D*_h_, nm	Δ*D*_h_, nm	PDI	ΔPDI	ζ‐pot	Δζ‐pot
TF‐DMPC:HIOTD (8.3:1.7)	140 ± 3	29 ± 3	0.10 ± 0.01	−0.02 ± 0.01	−35 ± 1	−28 ± 1
TF‐DMPC:chol:HIOTD (6.6:1.7:1.7)	127 ± 2	10 ± 2	0.04 ± 0.02	−0.05 ± 0.02	−20 ± 1	−14 ± 1
µF‐DMPC:HIOTD (8.3:1.7)	103 ± 5	−18 ± 5	0.4 ± 0.1	0.3 ± 0.1	0.6 ± 0.1	0.3 ± 1
µF‐DMPC:chol:HIOTD (6.6:1.7:1.7)	125 ± 5	21 ± 5	0.3 ± 0.1	0.2 ± 0.1	0.5 ± 0.1	0

**FIGURE 4:**
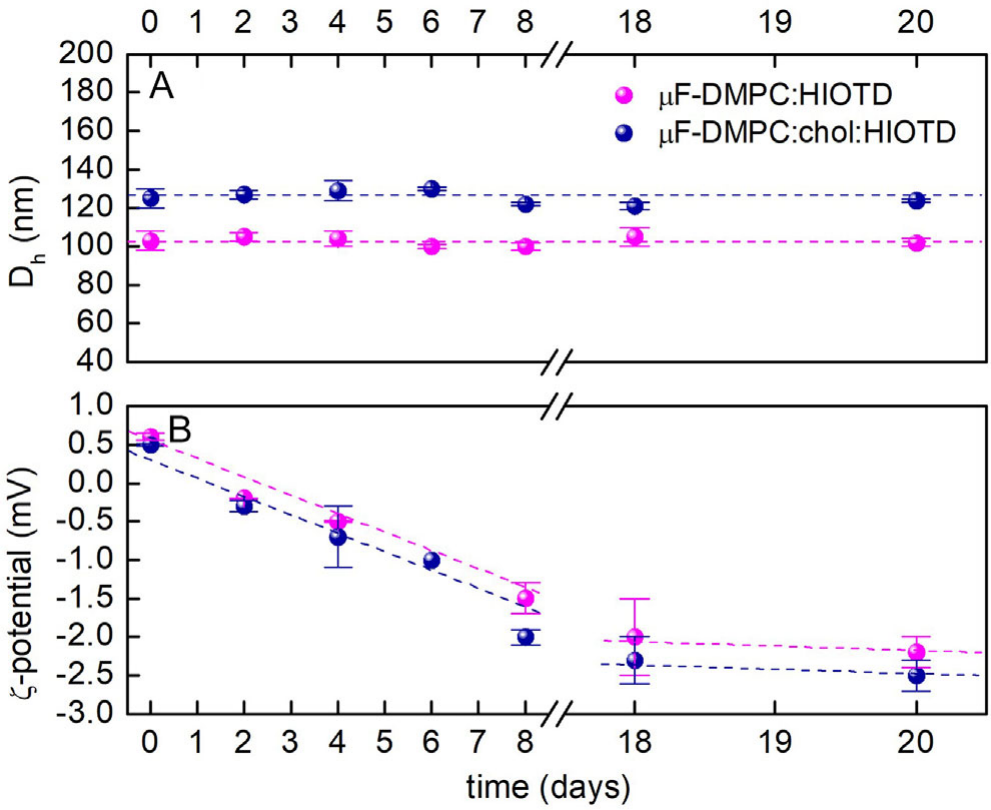
(A) Hydrodynamic diameter (*D*_h_) and (B) ζ‐potential values of µF‐HIOTD loaded liposomes, after dialysis. Error bar associated is the standard deviation of three repeated measurements.

Incubation with HIOTD produces very different effects in TF‐ and dialyzed µF‐liposomes. TF‐liposomes show significantly negative *ζ*‐potential values, amenable to the incorporated HIOTD molecule, whose negative charge derives from the acidic character of the oxime moiety of HIOTD. This molecule has a pKa of 8.7, so approximately 10% dissociation of the acidic groups is expected at pH 7.4 [33]. However, the zwitterionic nature of DMPC offers multiple sites for ionic interactions that may enhance the acidity of the oxime group within the confined environment at the liposome–water interface. On the other hand, *ζ*‐potential values for µF‐liposomes, which are close to neutrality to begin with, do not exhibit significant changes after HIOTD incubation. The PDI of µF‐liposomes undergoes a significant increase upon HIOTD incubation, with a large positive increment, whereas no effect is observed on the PDI of TF‐liposomes (Tables 1 and 2). Nevertheless, NNLS analysis of DLS measurements of µF‐DMPC:HIOTD and µF‐DMPC:chol:HIOTD shows that this increase is due to the presence of large objects of micrometric size, almost negligible in number (less than 1% in number‐weighted analysis), coexisting with the most abundant population of liposomes (Figure S3). As shown in Figure 4A, the *D*_h_ of HIOTD‐loaded µF‐liposomes remains stable for at least 20 days, in keeping with that of TF‐liposomes (data not shown), so the presence of the larger aggregates does not affect the colloidal stability of µF‐liposomes. On the other hand, *ζ*‐potential values of HIOTD µF‐liposomes gradually became slightly more negative (Figure 4B), until a constant value of ca. −2 mV was reached after 18 days. This evolution points to a slow equilibration of incorporated HIOTD toward an arrangement more similar to that of TF‐liposomes, following further release of ethanol from the bilayer over a longer timescale than that of the dialysis. However, the *ζ*‐potential value in *μ*F‐liposomes is more than ten times smaller than that of TF‐liposomes, which is consistent with a smaller amount of HIA group being exposed at the bilayer interface.

**FIGURE 5:**
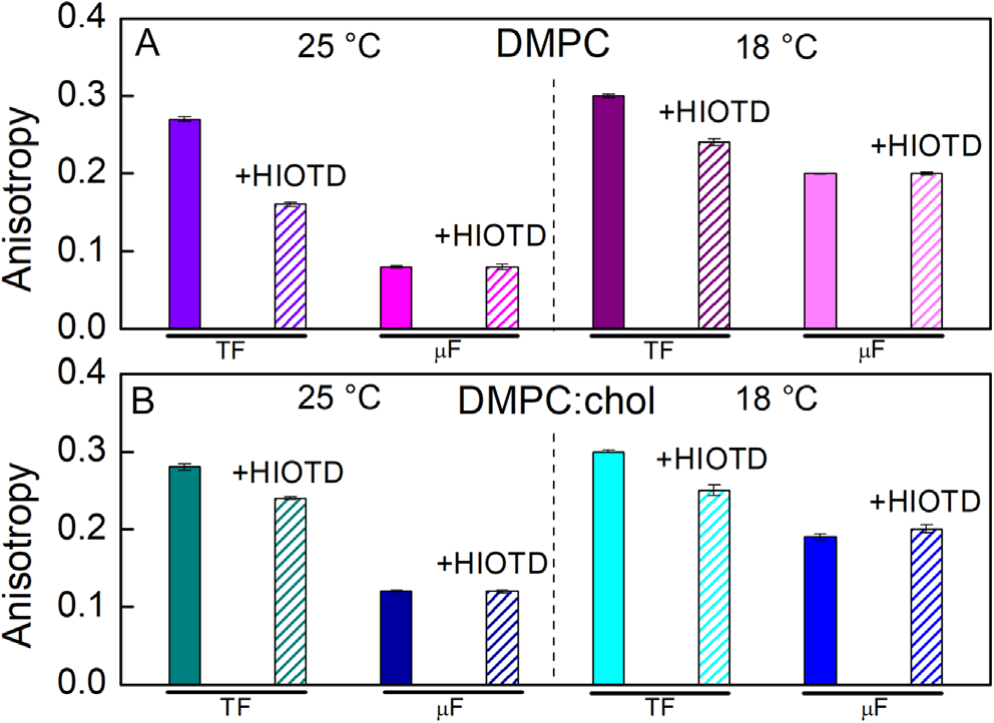
Fluorescence anisotropy for DPH probe in (A) TF‐DMPC and µF‐DMPC liposomes and (B) TF‐DMPC:chol and µF‐DMPC:chol liposomes, containing or not containing HIOTD (patterned and full bars, respectively), measured at two different temperatures.

The effect of loading of the HIOTD molecule in the lipid bilayer was also explored by fluorescence anisotropy measurements, performed at *T* = 25 °C, using the DPH probe, as previously described. As shown in Figure 5, both TF‐DMPC and TF‐DMPC:chol liposomes show a decrease in the anisotropy value after HIOTD loading. Overall, this suggests that the insertion of HIOTD mostly perturbs the hydrophobic region of the bilayer. Conversely, incubation of µF‐liposomes (DMPC and DMPC:chol) with HIOTD does not alter the anisotropy values, likely due to the predominant masking effect of ethanol (Figure 5). A similar behavior, even if less marked in its extent, is observed for the analysis performed at *T* = 18 °C, as reported in Figure 5. As already observed for plain liposomes, at this temperature the compaction of the bilayer prevails over the disordering effect caused by the HIOTD incorporation.

In summary, residual ethanol increases the fluidity of µF‐liposomes, enabling HIOTD incorporation without altering bilayer fluidity or the nearly neutral *ζ*‐potential. By contrast, TF‐liposomes exhibit a more negative *ζ*‐potential, likely as a consequence of oxime deprotonation at the interface.

### 2.3 Evaluation of the Metal Complexing Ability of HIOTD Incorporated in Liposomes

**FIGURE 6:**
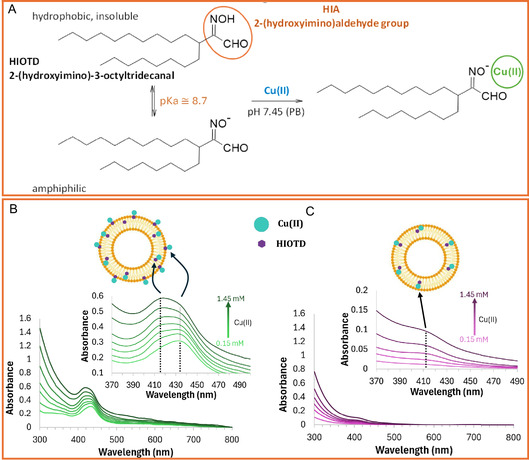
(A): Cu(II) complexation with 2‐(hydroxyimino)‐3‐octyltridecanal (HIOTD) molecule employed in this study. Hydrophobic HIOTD and its amphiphilic dissociated form are in equilibrium in phosphate buffer (PB) solution (0.01 M, pH 7.4). Cu(II) complexation occurs with deprotonation of the HIA group, the apparent pKa of the complex being lower than that of HIOTD. UV–vis spectra (quartz cell, 1 cm optical path) of (B) HIOTD TF‐liposomes and (C) HIOTD µF‐liposomes in PB. Cu(OAc)_2_ 0.15, 0.30, 0.45, 0.60, 0.90, 1.2, 1.45 mM. Cu(II)‐free spectra of the corresponding liposomal formulations with HIOTD were used as reference. In both insets, the dotted lines mark the position of the relevant spectroscopic features for clarity. Absorbance was zeroed at 800 nm to compensate for residual turbidity derived from Cu(II) in PB. In panels (B) and (C), a schematic representation of HIOTD localization in HIOTD TF‐ and µF‐liposomes, deduced from UV analysis, is shown.

HIOTD is a water‐insoluble molecule, and in this investigation its Cu(II) complexation in aqueous environments was studied for the first time. In the conditions used in the present study, the acidic dissociation of HIOTD, which has been shown to be essential for Cu(II) binding [35], should be promoted by the slightly alkaline pH of the PB, provided the HIA group is exposed to the aqueous environment (Figure 6A).

Results from DLS, DELS, DSC and fluorescence anisotropy described in the previous sections show that HIOTD is differently included in the TF‐ and *μ*F‐liposomes, mostly owing to residual ethanol in the bilayer of *μ*F‐liposomes. We anticipated that traces of alcohol may affect the positioning of HIOTD within the lipid bilayer, as well as the local solvation of the HIOTD‐Cu(II) complex and of Cu(II) as it approaches the ligand. For this reason, we resorted to UV–vis spectroscopy to highlight fine differences between HIOTD‐Cu(II) complex formation in liposomes obtained with the two methods. In fact, as shown in our previous work [35, 36], Cu(II)‐HIA complexes exhibit a sharp and intense LMCT band in the 450–400 nm region of the UV–vis spectrum (see Supporting Information for details) that has allowed us to determine the HIOTD/Cu = 1:1 stoichiometry through the method of continuous variations (Figure S4). It has also been shown that the presence of a base to deprotonate the oxime is necessary for the optimal complexation of Cu(II), and that the acidic constant of the HIA group decreases by several orders of magnitude upon Cu(II) complexation. The proton acceptor can be the acetate counterion or an added base.

Since DMPC and DMPC:chol liposomes incorporating HIOTD exhibit comparable behavior and physicochemical properties, the binding ability of Cu(II) was investigated only on the cholesterol‐free liposomal formulation. All experiments were run in PB, to which aliquots of a stock solution of Cu(OAc)_2_ in water were added. The stock solution was prepared in water due to poor solubility of Cu(II) in PB and to limit the impact of the additions on the ionic strength of the solutions. Preliminarily, blank experiments were run with HIOTD‐free DMPC liposomes and with a dispersion of HIOTD aggregates in PB (see Experimental for details in Figure S5).

No additional bands are detected upon addition of Cu(II) to HIOTD‐free liposomes (Figure S5), besides the expected increase of the absorbance at low wavelengths (<400 nm) amenable to solubilized Cu(II) and the baseline shift due to turbidity caused by insoluble copper salts. The typical broad band of copper ions in the visible region (about 700 nm) is below detection. Upon adding Cu(II) to the HIOTD aggregates, instead, a small band at 435 nm appears, indicating that, despite its hydrophobicity, a small fraction of HIOTD is brought into solution thanks to Cu(II) complexation in PB.

Figure 6B shows the spectra of HIOTD TF‐ and µF‐liposomes with increasing concentrations of Cu(II). The spectra of the corresponding copper‐free liposomes were subtracted as reference to best single out the effect of HIOTD‐Cu(II) complexation. At low Cu(II) concentration (Cu 0.15 mM, corresponding to half the concentration of HIOTD present in the system), TF‐liposomes exhibit a broad band with a maximum at about 435 nm and a shoulder around 415 nm (Figure 6A, light green curve). The intensity of the shoulder increases upon increasing Cu(II) concentration until the maximum of the band shifts to 415 nm (dark green curve) with a large excess of Cu(II) (Cu(II) > 1.2 mM, i.e., more than sixfold excess with respect to HIOTD concentration.

It should be noted that there is no evidence of residual HIOTD aggregates after incubation with TF‐liposomes, as shown in Figure S3. Thus, the 435 nm band cannot be ascribed to Cu(II) complexation occurring outside the TF‐liposomes, and it is inferred that the two overlapping bands in Figure 6A originate from Cu(II) complexation by HIOTD inserted in two distinct modes within the lipid bilayer. Specifically, it should be noted that the Cu(II)‐HIOTD complex in polar media (Figure S4, acetonitrile; Figure S5, PB) exhibits a maximum at 435 nm, whereas in the previous work on polymeric micelles the maximum of the Cu(II)‐HIA complex in the hydrophobic environment provided by the core of micelles was observed at 410–415 nm [35]. Figure S4 shows that this blueshift also results from forming the Cu(II)‐HIA complex with aqueous Cu(II) in ethyl acetate, i.e., a medium of low polarity. The extent of this shift is in keeping with the solvatochromic effect observed by other authors with Cu(II) complexes with Schiff bases [58]. On this basis, it can be speculated that the HIA group of a fraction of the HIOTD inserted in the lipid bilayer of the TF‐liposomes is exposed toward the polar bilayer surface, thus originating the 435 nm maximum early in the Cu(II) addition process. The remaining HIOTD would be inserted deeper in the hydrophobic part of the bilayer, so that the second band at lower wavelengths may be ascribed to complexation occurring when excess Cu(II) diffuses within the bilayer [51, 59]. Note that the overlap of the strong UV band of free Cu(II) (Figure S5) and of the two unresolved bands of the complex hampers the estimation of the binding constant for the complex within the TF‐ liposome. On a side note, the combination of HIOTD‐Cu(II) and TF liposomes constitutes an alternative to loading Cu(II) in the aqueous compartment or linking hydrophilic ligands to the liposome surface. Similar systems are actively investigated, for instance, as contrast agents with 64Cu, or for their anticancer or antibacterial effect, amenable to the well‐known Cu microbial toxicity [60, 62].

The spectra of *μ*F‐HIOTD liposomes complexed with Cu(II) shown in Figure 6C exhibit an overall lower intensity. Furthermore, in this case, the appearance of the 415 nm maximum (indeed of very low intensity) with a large excess of Cu(II) is not preceded by the 435 nm band. This result indicates that the small number of large aggregates (1%) described in the previous section after HIOTD incubation with *μ*F‐liposomes contains a negligible amount of HIOTD. In other words, the spectroscopic properties of the HIOTD‐Cu(II) system within *μ*F‐liposomes suggest a hydrophobic environment for the complex and provide no evidence for complexation in a polar aqueous environment. This result, combined with the nearly neutral value of the *ζ*‐potential of *μ*F‐DMPC:HIOTD liposomes (see Table 2), suggests that most of the HIOTD is inserted in the bilayer of the *μ*F‐liposomes, far from the ethanol‐contaminated bilayer surface. On these grounds, we suggest that, in keeping with the spectroscopic results observed with TF‐liposomes, the appearance of the weak 415 nm band is attributable to diffusion of excess Cu(II) within the hydrophobic part of the bilayer in *μ*F‐liposomes.

Unfortunately, the intensity of the 410 nm band of the complex is very small compared to the overlapping strong UV band of free Cu(II), making any attempt at determining the binding constant from UV–vis spectrometry unreliable. Furthermore, DLS analysis, used to monitor the effect of Cu(II) addition on the average size of the objects, indicated the presence of micrometric objects beyond a certain threshold concentration of added salt, which may be related to fusion among liposomes (Figure S6).

**FIGURE 7:**
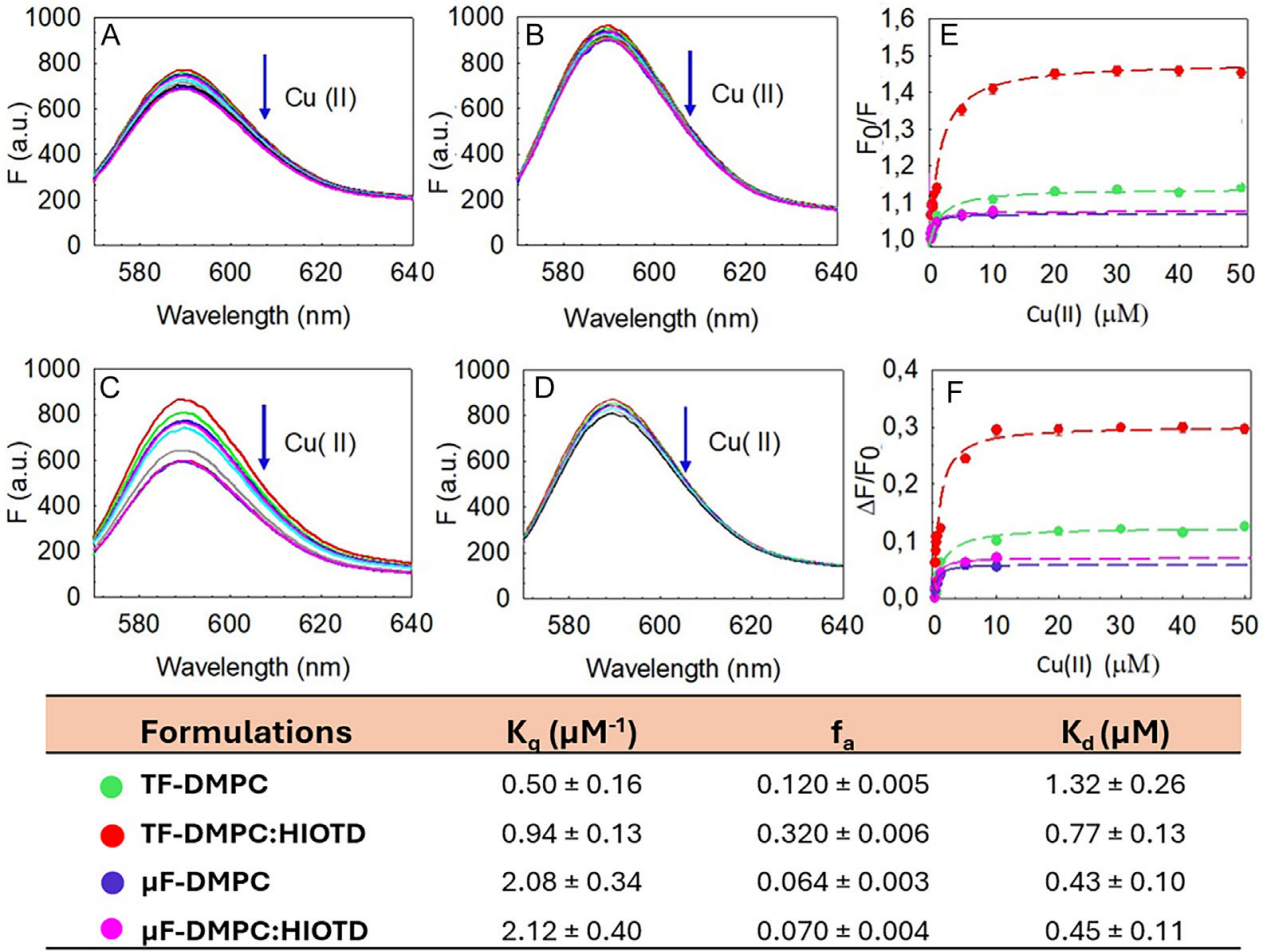
Fluorescence emission spectra of Rho‐labeled liposomes for the different formulations: (A) TF‐DMPC (60 in total lipids µM), (B) µF‐DMPC (30 µM in total lipids), (C) TF‐DMPC:HIOTD (60 µM in total lipids), and (D) µF‐DMPC:HIOTD liposomes (30 µM in total lipids), in the presence of different concentrations of Cu(II) (from 0.1  to 50 µM) in PB solution (0.01 M, pH 7.4). The vertical arrow indicates the increasing concentration of Cu(II). (E) Stern–Volmer plots and (F) Absorption isotherms of Rho‐PE‐embedded TF‐liposomes and µF‐liposomes in the presence of Cu(II), obtained by analyzing the spectra shown in panels A‐B‐C‐D. Symbols: TF‐DMPC (green circle), HIOTD TF‐DMPC (red circle), µF‐DMPC (blue circle), µF‐DMPC:HIOTD (purple circle). Quenching constant *K*_q_ and accessible fraction fa of Rho‐PE molecules embedded in TF‐liposomes and µF‐liposomes, obtained by fitting the fluorescence quenching data by the modified Stern–Volmer equation (Equation (1)), and apparent Cu(II) dissociation constant *K*_d_ by liposomes (from Equation (2)) are reported in the table.

After demonstrating the metal‐binding ability of the HIOTD‐loaded liposomes, the strong quenching of Cu(II) was exploited to indirectly estimate the binding affinity between this metal and the liposomes as a function of composition and preparation method [63]. To this end, a lipid functionalized at the hydrophilic head with Rhodamine (Rho) was added to the lipid mixture at the beginning of liposome preparation in very small amounts (0.2%), without altering their physicochemical properties. Rhodamine fluorescence is known to be quenched by Cu(II) through the energy transfer mechanism. Fluorescence quenching occurs only when Cu(II) and Rhodamine come into contact. Therefore, the quenching phenomenon becomes more efficient when Cu(II) is localized near the fluorophore. Rhodamine‐labeled µF‐ or TF‐liposomes (Rho‐labeled liposomes), with and without HIOTD, were titrated with a Cu(II) solution (Figure 7). The total lipid concentrations in the quenching experiment are lower than those used for UV titration, for reasons intrinsically related to the different technique.

As shown in Figure 7A,B, even at the highest Cu(II) concentration tested (50 *μ*M), only negligible quenching was observed for DMPC liposomes, produced by both methods, as expected. DMPC is formally a neutral lipid, and therefore it can bind copper ions only through weak electrostatic interactions, involving its negatively charged phosphate group. Indeed, it has been previously reported that PC exhibits a poor ability to bind copper compared with other lipids, such as PS and PE, which can form stable complexes with Cu(II). In contrast, in the same Cu(II) concentration range, a more pronounced quenching was observed in the HIOTD‐containing liposomes, as can be expected through specific chelation by HIOTD in the outer leaflet of liposomes (Figure 7C,D).

To highlight any quantitative differences in the quenching ability of Cu(II), the spectra discussed above can be elaborated by two different approaches. The most immediate and common approach is to use the Stern–Volmer equation, which allows for studying the efficiency of Rhodamine quenching by Cu(II) and the accessibility of the fluorophore to the quencher; this is the most direct because the achievable parameters (quenching constant, *K*_q_, and accessible fluorophore fraction, *f*_a_) are closely related to the physical phenomenon observed, i.e., fluorophore quenching. The second approach involves data processing by using a binding equation to obtain a quantitative indicator of Cu(II) affinity to liposomes, the binding dissociation constant, *K*_d_. This parameter is obtained indirectly, because the phenomenon of fluorescence quenching is ascribed to the interaction of Cu(II) with the fluorophore and is not a direct measure of Cu(II) binding to HIOTD‐lipid. Both approaches were followed to check results consistency and therefore reinforce the reliability of the obtained *K*_d_.

First, the ratio *F*_0_ */F* between the Rho fluorescence intensity measured in the absence of Cu(II) (*F*_0_) and the one measured at different Cu(II) concentrations (*F*) was plotted as a function of Cu(II) concentration. In this representation, a nonlinear trend is immediately evident (Figure 7E). This behavior suggests that two different populations of Rho coexist in the liposome bilayer, the first one consisting of a fraction of molecules that are easily accessible to the Cu(II) ions (*f*_a_), characterized by a specific quenching constant (*K*_q_), and the second one of molecules (1*– f*_a_) that never come into contact with the quencher. This is reasonable considering the different localization of Rho lipids in the vesicles, where the ones in the inner leaflet of the bilayer do not come in contact with Cu(II). By fitting the experimental data with the Stern–Volmer equation modified for the presence of two fluorophore populations [64]

(1) $$\frac{F_{0}}{F}=\frac{1+K_{q}[\mathrm{Cu}(\mathrm{II})]}{1+\left(1-f_{a}\right)K_{q}[\mathrm{Cu}(\mathrm{II})]}$$

the *K*_q_ and *f*_a_ values for each liposome formulation are obtained (see bottom Table in Figure 7).

The quenching constant *K*_q_ is a measure of the Cu(II)‐Rho complex stability, and its value depends on the accessibility of the fluorophore to the copper ions, in particular, on the separation distance within the excited‐state complex, which is influenced by diffusion and steric shielding of the fluorophore. In the case of µF‐liposomes, the higher *K*_q_ values may seem counterintuitive, considering the lower quenching efficiency highlighted in the spectra in Figure 7. Actually, they may be explained by considering the higher fluidity of the bilayer experienced by Rho and Rho‐Cu(II) complex in µF‐liposomes, caused by the presence of ethanol, as pointed out by fluorescence anisotropy experiments.

Interestingly, the plots of µF‐liposomes, and consequently, the corresponding *K*_q_ and *f*_a_ values, show only slight differences, independently of the incorporation of HIOTD. This effect may result from HIOTD being positioned within the hydrophobic part of the bilayer and away from the polar interface, a possibility already suggested by the *ζ*‐potential data (Table 2) and further supported by the UV–vis results (Figure 6C), which may limit its accessibility toward Cu(II).

The quenching behavior of TF‐liposomes, on the other hand, is strongly influenced by the presence of HIOTD. In fact, TF‐liposomes containing HIOTD show both a higher quenching constant (*K*_q_ ∼ 0.94) and a larger fraction of Rho accessible to the quencher Cu(II) (*f*_a_ ∼ 0.320 ± 0.006), with respect to HIOTD‐free DMPC TF‐liposomes. The higher values of *K*_q_ and *f*_a_ indirectly confirm that HIOTD is able to form stable Cu(II) complexes on the liposome surface, influencing, in turn, the stability of the Rho‐Cu(II) complex. Once again, this hypothesis is supported by the UV–vis analysis (Figure 6B). Furthermore, the observed differences also suggest that the Cu(II)‐HIOTD complex remains anchored in the lipid bilayer: if this were not the case, fluorescence quenching of the Rho, which is bound to the liposome, would not be observed.

To obtain a quantitative parameter on the binding affinity between Cu(II) and the liposomes, the data obtained from the fluorescence quenching experiment can be also processed by plotting, as a function of Cu(II) concentration, the normalized quenched fraction of Rho, defined as Δ*F/F*_0_, where Δ*F* is the difference between fluorescence intensity of Rho in the absence of Cu(II) and quenched Rho, at each Cu(II) concentration, and *F*_0_ is the fluorescence of Rho in the absence of Cu(II). The obtained binding isotherms (Figure 7F) can be fitted by the following equation [65]:

(2) $$\frac{\Delta F}{F_{0}}=\frac{\Delta F_{\max}\max\left[\mathrm{Cu}\left(\mathrm{II}\right)\right]}{F_{0}\left(K_{d}+\left[\mathrm{Cu}\left(\mathrm{II}\right)\right]\right)}$$

where Δ*F*_max_ represents the maximum Rho fraction quenched at the saturation concentration, and *K*_d_ corresponds to the dissociation constant (see bottom Table in Figure 7).

In TF‐liposomes, the presence of HIOTD causes a significant difference: at 10 µM Cu(II), ∼10% of Rho quenching is observed in plain DMPC liposomes (Figure 7A), while in HIOTD‐liposomes the Rho quenching increases by a factor of 3, reaching ∼30% (Figure 7C). This evidence is also reflected in a lower *K*_d_ value for HIOTD‐loaded liposomes (∼0.77 ± 0.13 µM) with respect to DMPC (*K*_d_ ∼ 1.32 ± 0.26 µM), indicating a higher concentration of Cu(II) bound on the liposome surface. As already suggested by the UV spectroscopic signature, this evidence indirectly confirms that the binding moiety of the HIOTD molecule is well exposed to the surface and accessible to Cu(II), so it can act as a good metal ligand.

In the case of µF‐liposomes, the Rho‐quenching is low for both plain DMPC (∼4%, Figure 7C) and HIOTD liposomes (∼6%, Figure 7D), with similar *K*_*d*_ values (∼0.43−0.45 µM). This suggests a reduced interaction with Cu(II) in all µF‐liposomes, independent of the presence of HIOTD. The different quenching behavior of Rho in µF‐ or TF‐liposomes could likewise be attributed to the presence of residual ethanol in the bilayer of µF‐liposomes. In addition to the modification of the interfacial organization of lipids and the overall surface charge of liposomes, residual ethanol may have a role in inhibiting the nonspecific binding of copper ions with the lipid phosphate groups in plain µF‐DMPC liposomes, in the deprotonation of oxime and/or in the positioning of HIOTD in the double layer, thus reducing the effective concentration available for binding copper ions in µF‐DMPC:HIOTD liposomes.

Based on these results, the capability to interact with copper ions in the presence of competitors, such as EDTA, was investigated only on the TF‐liposomes, given the weak interaction of µF‐liposomes with Cu(II).

**FIGURE 8:**
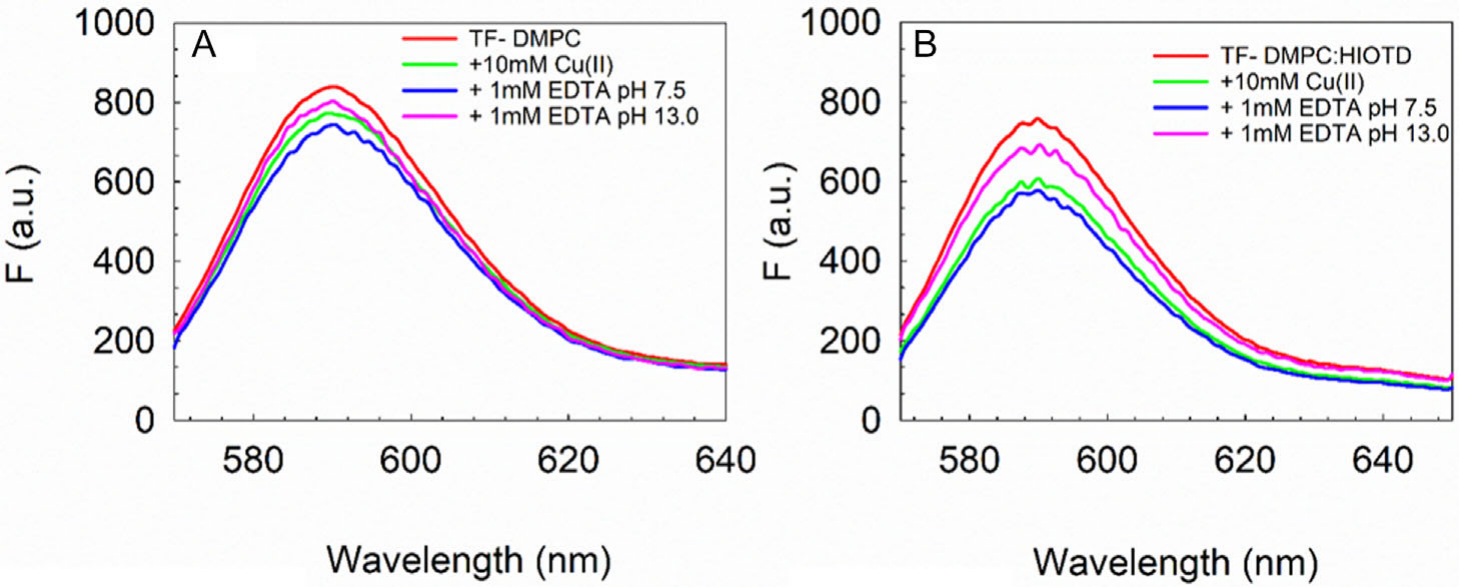
Fluorescence emission spectra of Rho‐labeled (A) TF‐DMPC:Cu(II) complex (60 µM:10 µM) and (B) TF‐DMPC:HIOTD‐Cu(II) complex (60 µM:10 µM), in PB solution (0.01 M, pH 7.4) after adding EDTA at different pH (13.0, 7.5).

Therefore, a competition experiment was performed by adding EDTA to Rho‐labeled TF‐DMPC liposomes loaded with Cu(II) or Cu(II)‐HIOTD complexes in PB solutions, at two different pH values (Figure 8; pH 7.5 and 13.0). Slight variations in the fluorescence emission spectra of Rho‐labeled TF‐DMPC liposomes with Cu(II) are observed. On the other hand, in the case of DMPC:HIOTD liposomes, the effect of EDTA is more pronounced at basic pH, resulting in an increase in fluorescence intensity, indicating that at pH 13.0 Cu(II) binding to Rho‐labeled TF‐DMPC:HIOTD liposomes is reversible.

Remarkably, instead, at physiological pH in the presence of EDTA, the fluorescence intensity of TF‐DMPC:HIOTD remains unaltered. These results suggest that in a physiological environment, even in the presence of a competitor, the presence of HIOTD on the surface of liposomes allows copper ions to be strongly chelated thanks to the favorable pK_a_ of HIA‐Cu(II) complexes [35].

## 3 Conclusions

This study highlights the influence of the preparation protocol of liposomes on their colloidal, structural, and functional properties by focusing attention on two different procedures, known as thin film preparation, followed by rehydration and extrusion (TF), and *μ*F. Each procedure requires the solubilization of lipids in a proper organic solvent, whose fate is determined by the subsequent steps of the two techniques. In the TF protocol, the solvent is completely evaporated prior to vesicle formation to allow the formation of a thin lipid film, whereas in the µF procedure, vesicle formation occurs in a mixture of aqueous solvent and alcohol, whose proportion is determined by the parameters used to mix the fluids. In µF protocols, solvent removal is usually introduced as a post‐processing step to achieve structurally stable, functionally active liposomes for advanced molecular and biomedical applications. However, residual solvent traces may still be present in the bilayer after purification, with a possible effect on the overall critical quality attributes of liposomes [23]. Here, for the first time to our knowledge, the role of ethanol traces in relation to the interaction of µF‐liposomes with a functional molecule has been evaluated, namely the metal‐complexing lipid HIOTD.

The investigation of size, *ζ*‐potential, fluorescence anisotropy, and melting temperature of µF‐liposomes shows that residual ethanol retained in the bilayer after dialysis does not compromise liposome colloidal stability, whereas it does affect lipid packing compared with TF‐liposomes.

Furthermore, upon incorporation of metal‐free HIOTD, TF‐liposomes display negative *ζ*‐potential due to oxime deprotonation at the interface, whereas µF‐liposomes exhibit almost neutral surface charges, hinting at a different arrangement and local environment in the µF‐and TF‐liposomes. Finally, when the function of the encapsulated HIOTD (i.e., Cu(II) ligation) is assessed, higher affinity and accessibility are found in TF‐ than in µF‐liposomes. Being sensitive to residual ethanol, the Cu(II)‐HIOTD system may provide a convenient tool to routinely probe the internal structure of lipid bilayers through simple and highly accessible UV–vis spectroscopy.

Our findings highlight how the choice of the proper preparation method may be instrumental to tailoring the properties of liposomes to the therapeutic target by impacting the complex set of molecular interactions that govern their structure‐function behavior. For instance, due to their higher deformability connected to the presence of residual ethanol, µF‐liposomes are preferred for dermal delivery, with respect to TF‐liposomes [66]. Hydrophobic Cu(II)‐HIOTD complex, designed to be a good fit for the bilayer of liposomes, complements existing hydrophilic copper complexing systems suited for the surface modification of liposomes, such as DOTA or low molecular weight polyethyleneimine. Finally, should liposomal encapsulation of metal‐HIOTD complexes be considered for applications in theranostics or as antimicrobial or anticancer nanomaterials, our results suggest that the conventional TF preparation method grants superior performance in terms of metal loading and accessibility.

## 4 Experimental Section

### 4.1 Materials

The lipids 1,2‐dimyristoyl‐*sn*‐glycero‐3‐phosphocholine (DMPC) (purity > 99%), 1,2‐dimyristoyl‐*sn*‐glycero‐3‐phosphoethanolamine‐N‐(lissamine rhodamine B sulfonyl) (ammonium salt) (14:00 Liss Rhod PE) (purity > 99%) were purchased from Avanti Polar Lipids, Inc. (Alabaster, AL, USA) and used as received. Cholesterol (chol) (purity > 99%), hydrochloric acid, sodium hydroxide, copper(II) acetate 98%, ethanol (absolute for analysis EMSURE ACS, ISO, Reag. Ph Eur), sodium phosphate dibasic (Na_2_HPO_4_), sodium phosphate monobasic (NaH_2_PO_4_), diphenylhexatriene (DPH), cellulose dialysis membranes (14,000 Da cut off) were purchased from Sigma–Aldrich (St. Louis, MO, USA). All reagents were analytical grade and used without further purification. In all cases, bi‐distilled water (Merck KGaA, Darmstadt, Germany) was used in the preparation of solutions.

#### 4.1.1 Synthesis of 2‐(hydroxyimino)‐3‐Octyltridecanal (HIOTD)

2‐(hydroxyimino)‐3‐octyltridecanal was obtained from 2‐octyldodecan‐1‐ol with a three‐step synthesis as previously described [33].

**2‐octyldodecanal.** 2‐octyldodecan‐1‐ol was submitted to Parikh–Doering oxidation. Briefly, the alcohol (925 mg, 3.10 mmol) was reacted in 20 mL CH_2_Cl_2_ and 4.0 equiv Et_3_N with 3.0 equiv SO_3_·Pyridine complex previously dissolved in DMSO. The crude product was purified by silica gel chromatography to yield 2‐octyldodecanal (colorless liquid 830 mg, 2.80 mmol, 90% yield).

**3‐octyltridecanal.** 2‐octyldodecanal was homologated to 3‐octyltridecanal by Wittig reaction. 2‐octyldodecanal (0.83 g, 2.8 mmol) was reacted in anhydrous THF with CH_3_OCH_2_PPh_3_Cl (2.9 g, 8.3 mmol) and *t*‐BuOK (940 mg, 8.4 mmol). After careful workup to thoroughly remove triphenylphosphine oxide, the product was purified by silica gel chromatography. White solid, 0.35 g (1.1 mmol), 40% yield.

**2‐(hydroxyimino)‐3‐octyltridecanal.** The α‐oximation reaction was performed on 350 mg (1.03 mmol) of 3‐octyltridecanal in DMF with pyrrolidine (0.6 mmol, 0.2 equiv), p‐toluenesulfonic acid (0.6 mmol, 0.2 equiv), NaNO_2_ (3.0 mmol, 1.0 equiv), and FeCl_3_ · 6H_2_O (3.0 mmol, 1.0 equiv). Reaction completion was monitored by TLC. After workup, the crude product was purified by flash chromatography with a mixture of hexane/Et_2_O in a ratio of 90:1. Gray solid, 210 mg (0.62 mmol), 60% yield. 1H‐NMR (300 MHz, DMSO‐d6) δ: 12.8 (s, 1H); 9.34 (s, 1H); 3.06 (m, 1H); 1.68 (m, 2H); 1.46 (m, 2H); 1.19 (m, 28H), 0.839 (t, J = 6.5 Hz, 6H). 13C{1H}‐NMR (75 MHz, DMSO‐d6) δ 192.2; 160.8; 34.4; 31.3; 31.3; 30.7; 29.0; 28.9; 28.9; 28.7; 28.7; 27.3; 22.12; 22.1; 13.9.

### 4.2 Preparation of Liposomes

#### 4.2.1 Production of Liposomes by Thin Film Hydration and Extrusion (TF‐Liposomes)

Aqueous dispersions of empty liposomes were prepared according to a reported procedure [67]. A lipid film was prepared on the inside wall of a round‐bottom flask by evaporation of CHCl_3_ solution containing the proper amount of DMPC or DMPC:chol mixture to obtain a lipid DMPC:chol ratio of 9.5:0.5. The films obtained were stored overnight under reduced pressure (0.4 mbar), Then a proper amount of PB solution (0.01 M, pH 7.4) was added to the lipid film to obtain a 1 or 2 mM lipid dispersion. The dispersions were vortex‐mixed and then freeze−thawed six times from liquid nitrogen to 313 K and then extruded (10 times) by a 10 mL extruder (Lipex Biomembranes, Vancouver, CA) equipped with a 100 nm polycarbonate membrane (Merck KGaA, Darmstadt, Germany). The extrusions were carried out well above the transition temperature of liposomes.

#### 4.2.2 Production of Liposomes by Microfluidics (µF‐Liposomes)

Liposomes were prepared by the Dolomite *μ*F system (Royston, UK), following the protocol reported in the literature. In this system, the two phases, organic and aqueous, are fed from two different reservoirs, each equipped with pressure‐driven pumps and an in‐line Mitos flow sensor (0.2–5 ml/min), allowing the accurate regulation of the desired flow rates. A starting calibration was carried out to give accurate flow rate readings. A glass Micromixer Chip was used for rapid mixing of two fluid streams. As for TF‐liposomes, two different formulations were prepared, pure DMPC and mixed DMPC:chol (DMPC:chol molar ratio = 9.5:0.5). The lipid mixture (10 mM initial concentration) was dissolved in ethanol (organic phase) and injected into the center channel of the microfluidic device. PB solution (0.01 M, pH 7.4) (aqueous phase) was injected into two lateral channels intersecting with the central one. The chosen FRR, defined as the ratio between the aqueous and organic phase flow rate, was 12; while the total flow rate (TFR), defined as the total speed at which both fluid streams are pumped through the two separate inlets, was 0.65 mL min^−1^. In the final flow, the concentration of ethanol was 8%v/v and that of liposomes was 1 mM. FRR and TFR parameters were chosen as the result of a systematic investigation addressed to achieve liposomes of size around 100 nm and low polydispersity, for a better comparison with TF‐liposomes. All samples were prepared at room temperature and collected in plastic microtubes. To remove ethanol, µF‐liposomes were dialyzed immediately after preparation against PB solution (0.01 M, pH 7.4) (1 mL of liposomes in 250 mL of PB) using dialysis tubes (14,000 Da cut‐off) at 25 °C for 24 h. The area of the dialysis membrane, separating the liposomes from the PB, is approximately 1 cm^2^.

#### 4.2.3 Incorporation of HIOTD in Liposomes

In order to obtain liposomes with the HIOTD lipid, a proper volume of liposomes (TF‐ or µF‐) was incubated with dried 2‐(hydroxyimino)‐3‐octyltridecanal (HIOTD) to get the desired final lipid:HIOTD molar ratio (8.3:1.7), under stirring, at 37 °C for 90 min. This protocol was chosen after a series of preliminary experiments aimed to evaluate the effectiveness of different incorporation protocols (data not shown).

### 4.3 Characterization of Liposomes

#### 4.3.1 Dynamic and Dielectrophoretic Light Scattering

Dynamic and dielectrophoretic light scattering (DLS, DELS) measurements for determination of size and *ζ*‐potential, respectively, were performed by the Nano Zetasizer ZS instrument (Malvern Instrument, UK), equipped with a polarized monochromatic beam emitted by a solid‐state laser (100 mW; *λ *= 642 nm) and a thermostated cell holder. For DLS measurement, scattered light was collected at an angle ϴ=173° (backscattering configuration). The autocorrelation functions have been first analyzed by the cumulant method to get the average hydrodynamic size and the polydispersity index (PDI) of the samples, since this is the most direct way for determination of size [68]. When PDI values are larger than 0.2–0.3, this analysis may not be significant because the size distribution could show more than one maximum. To ascertain this point, a volume‐weighted NNLS algorithm has been used in the framework of the Mie scattering theory dispersion to determine the whole size distribution [69]. For this analysis, the refractive index of liposomes has been used.

DELS measurements for determination of *ζ*‐potential of liposomes have been performed by means of the laser doppler microelectrophoresis technique implemented with the M3‐Phase Analysis Light Scattering (PALS) for a sensitive detection of the Doppler shift [70]. The electrophoretic mobility *μ* of the diffusing objects is then converted into a *ζ*‐potential using the Smoluchowski relation *ζ* = *μ* *η*/*ε*, where *ε* and *η* are the permittivity and the viscosity, respectively, of the solution. Temperature was fixed to 25 °C in all the measurements.

Conductivity of the samples has been measured by a Nano Zetasizer ZS instrument (Malvern Instrument, UK), simultaneously to the determination of the *ζ*‐potential.

#### 4.3.2 Differential Scanning Calorimetry

The heat capacity profiles of the liposomes were measured using a nano‐DSC instrument from TA Instruments (New Castle, USA), equipped with two gold capillary cells, each with a volume of 300 µL. The total lipid concentration in each sample was between 0.8 and 2.5 mM. The temperature range for the experiments was set between 0 and 50 °C, with a scanning rate of 1 °C per minute. The capillary cells were maintained at a pressure of 3 atm to prevent bubble formation during the scans. A baseline scan using buffer only was recorded under the same conditions and subtracted from the sample scans before peak analysis. For each sample, at least four heating and cooling cycles were performed to confirm the reproducibility and reversibility of the results. For each experiment, three technical and two experimental replicates were performed, and the average enthalpy change (Δ*H*°_m_) and melting temperature (*T*_m_) were reported. The standard deviations were always lower than 10% of the obtained values.

#### 4.3.3 Fluorescence Anisotropy

Fluidity of liposomal bilayers has been evaluated by using diphenylhexatriene (DPH) as a probe. 50 *μ*L of fluorescent probe (0.2 mM) was added to lipid mixtures (ratio probe:lipids = 1:2000) in the first step of vesicle preparation, and probe‐loaded vesicles were prepared. An LS55 spectrofluorimeter (PerkinElmer, USA) was used to measure the fluorescence anisotropy at 25 °C, defined as A = (*I*_vv_ − *GI*_vh_ )*/*(*I*_vv_ + 2*GI*_vh_), where *I*_vv_ and *I*_vh_ are the intensities of the emitted fluorescence parallel and perpendicular to the direction of the vertically polarized excitation light, respectively, and *G* = *I*_vh_/*I*_hh_ is a correction factor [53, 71, 72]. The fluorescence anisotropy was recorded at 25 °C. The fluorescence anisotropy values are inversely proportional to the membrane fluidity, and high values correspond to a high structural order and/or a large viscosity of the membrane [53, 72].

#### 4.3.4 UV–Vis Absorption Spectroscopy

UV–vis absorption spectroscopy was used to evaluate the formation of Cu(II)‐HIOTD complexes. Spectra (800 – 300 nm) were recorded with a Cary 3500 Compact UV–vis spectrophotometer (Agilent, USA) at 25 °C with a scan rate of 300 nm min^−1^ using a 1 mL quartz cuvette of 1 cm optical path length. 5–10 *μ*L aliquots of a 20 mM Cu(OAc)_2_ stock solution were added to 0.650 mL liposome dispersions in phosphate buffer (PB, 0.01 M, pH 7.4) at a total initial lipid concentration of 2 mM, and spectra were recorded after each addition. Due to the limited solubility of Cu(OAc)_2_ in PB, the stock solution had been prepared in double‐distilled water. After each addition, the dispersion was gently stirred for 5 min, followed by 2–3 min waiting before measurement to allow the settling of any insoluble excess copper salts. Residual turbidity was compensated by zeroing the reading at 800 nm. The Cu(II) concentrations in the cuvette were 0.15–1.70 mM, and the final volume of the Cu(II)‐liposomes mixture after all additions was 0.710 mL. PB was used as a reference to record the spectra of HIOTD‐free liposomes at increasing Cu(II) concentrations. The spectra of copper‐free HIOTD liposomes were subtracted from the spectra of the same liposomes with added Cu(II).

In a different set of experiments, HIOTD 11.5 mg (34 mmol) was suspended in 1 mL PB, stirred for 90 min at 37 °C, then 0.650 mL supernatant was transferred to the quartz cuvette, and aliquots of Cu(OAc)_2_ stock solution were added as described above. PB was used as reference in this case.

#### 4.3.5 Fluorescence Quenching Experiments

Fluorescence quenching experiments were carried out on liposomes, labeled with the fluorescent lipid 1,2‐dimyristoyl‐*sn*‐glycero‐3‐phosphoethanolamine‐N‐(lissamine rhodamine B sulfonyl) (ammonium salt), to estimate the binding affinity between them and Cu(II). The fluorescent lipid, hereafter referred to as Rho, was added to lipid mixtures in organic solvent (0.2% mol) in the first step of liposome preparation. When applicable, HIOTD was inserted through incubation with the pre‐formed liposomes, as described for Rho‐free liposomes. Aliquots of a Cu(OAc)_2_ stock solution (0.1  or 1 mM) in double‐distilled water were added to Rho‐liposomes diluted in PB buffer to 30  or 60 µM in total lipids, for µF and TF‐liposomes, respectively, to get a Cu(II) concentration in the interval 0–50 µM. After each Cu(II) addition, fluorescence emission spectra were recorded using a LS55 spectrofluorimeter (PerkinElmer, USA) in the range 570–650 nm at 25 °C (*λ*_exc_: 560 nm; *λ*_em_max_: 592 nm). At the end of the titration, since the experiment was conducted in the absence of an ionic strength buffer, the integrity of the liposomes and the stability of their diameter were verified by DLS measurements.

## Funding

This work was supported by Ministero dell'Università e della Ricerca (CUP B53D23013710006, CUP B53C20040570005).

## Conflicts of Interest

The authors declare no conflicts of interest.

## Supporting information

Supplementary Material

## Data Availability

The data that support the findings of this study are available on request from the corresponding author.
