# Supplementary material for: A Copper‐Complexing Lipid Unmasks the Ethanol Hidden Within Liposomes and Highlights the Relevance of Fabrication Method on the Performance of Lipophilic Functional Molecules
Source: Small Sci. 2026 Aug 4;6(8):e70357. doi: 10.1002/smsc.70357 (PMC13459806; doi:10.1002/smsc.70357)
Supplement: Supplementary file 1 [file SMSC-6-e70357-s001.pdf]

Supplementary Information

A Copper-Complexing Lipid Unmasks the Ethanol Hidden Within Liposomes and Highlights the Relevance of Fabrication Method on the Performance of Lipophilic Functional Molecules

Leonardo Severini[1#], Beatrice Simonis[2#], Davide Lanera[3§], Marco Campanile[4], Pompea Del Vecchio[4], Federica Di Mascolo[2,3], Isabella Ossani[2], Francesca D'Acunzo[2*], Cecilia Bombelli[2], Simona Sennato[1*]

[1]Institute for Complex Systems (ISC), National Research Council (CNR), c/o Department of Physics, Sapienza University of Rome, Piazzale Aldo Moro 5, 00185 Rome, Italy
[2]Institute for Biological Systems (ISB), National Research Council (CNR), Secondary Office of Rome-Reaction Mechanisms, c/o Department of Chemistry, Sapienza University of Rome, Piazzale Aldo Moro 5, 00185, Rome, Italy
[3]Department of Chemistry, Sapienza University of Rome, Piazzale Aldo Moro 5, 00185, Rome, Italy
[4]Department of Chemical Sciences, University of Naples Federico II, Via Cintia 26, 80126, Naples, Italy

# These authors contributed equally
§ present affiliation: University of Rome Tor Vergata, Rome, Italy
*Corresponding authors: francesca.dacunzo@cnr.it (Francesca D'Acunzo); simona.sennato@cnr.it (Simona Sennato)

1. Characterization of liposomes: NNLS size distribution analysis of DLS data, conductivity data and DSC curves

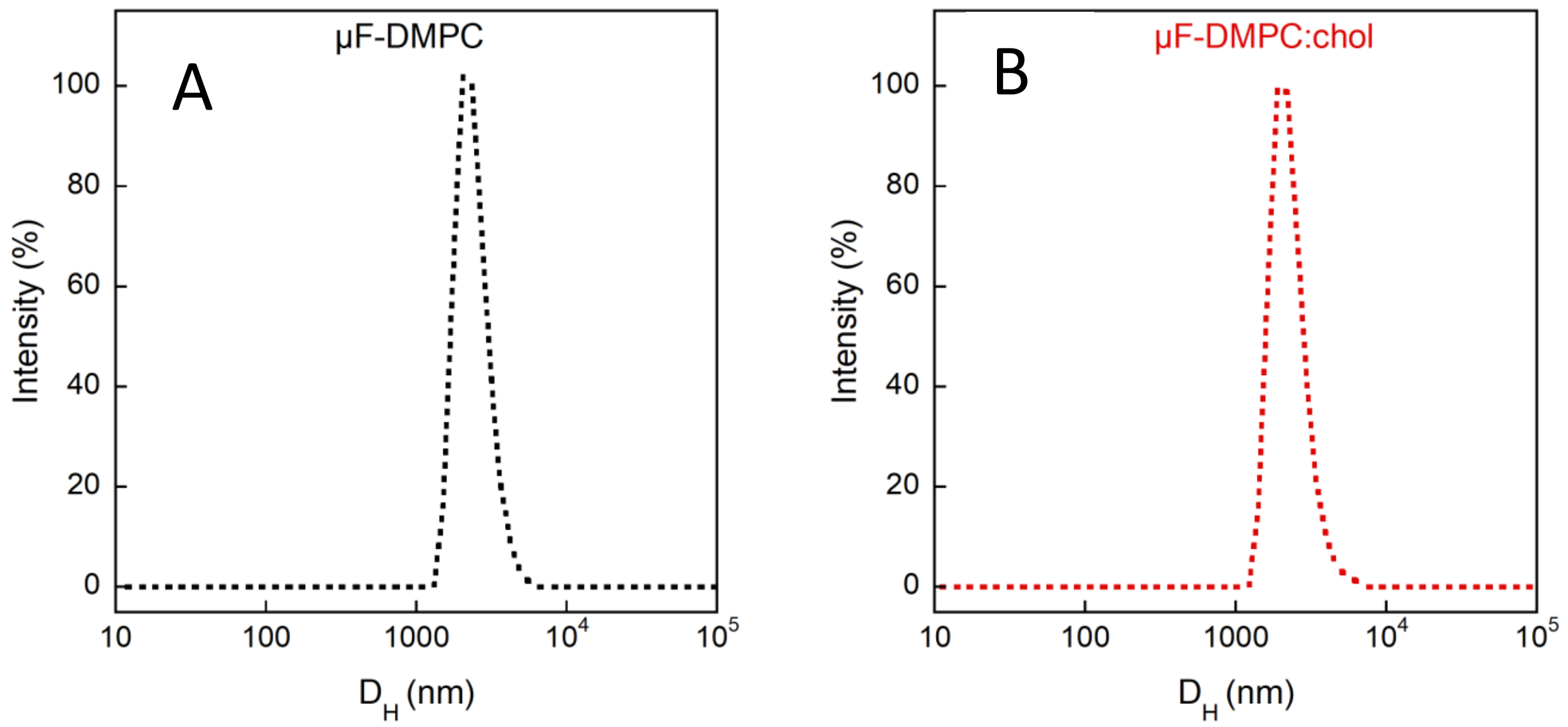

Figure S1. Intensity-weighted size distribution of undyalized µF-DMPC (**panel A**) and µF-DMPC:chol (**panel B**) liposomes, black and red profile respectively, measured 4 days after the preparation, in the aggregation regime.

Table S1. Average conductivity values, presented as mean ± SD (n = 3), of TF- and µF-liposomes, produced according to the indicated protocol.

**Sample (mole ratio)**	**Protocol**	**Conductivity (mS/cm)**
TF-DMPC	TF	3.9±0.1
TF- DMPC:chol (9.5:0.5)	TF	3.7±0.3
µF-DMPC	µF	0.9±0.2
µF-DMPC:chol (9.5:0.5)	µF	1.0±0.3
µF-DMPC	µF+dialysis	3.5±0.2
µF-DMPC:chol (9.5:0.5)	µF+dialysis	3.4±0.3

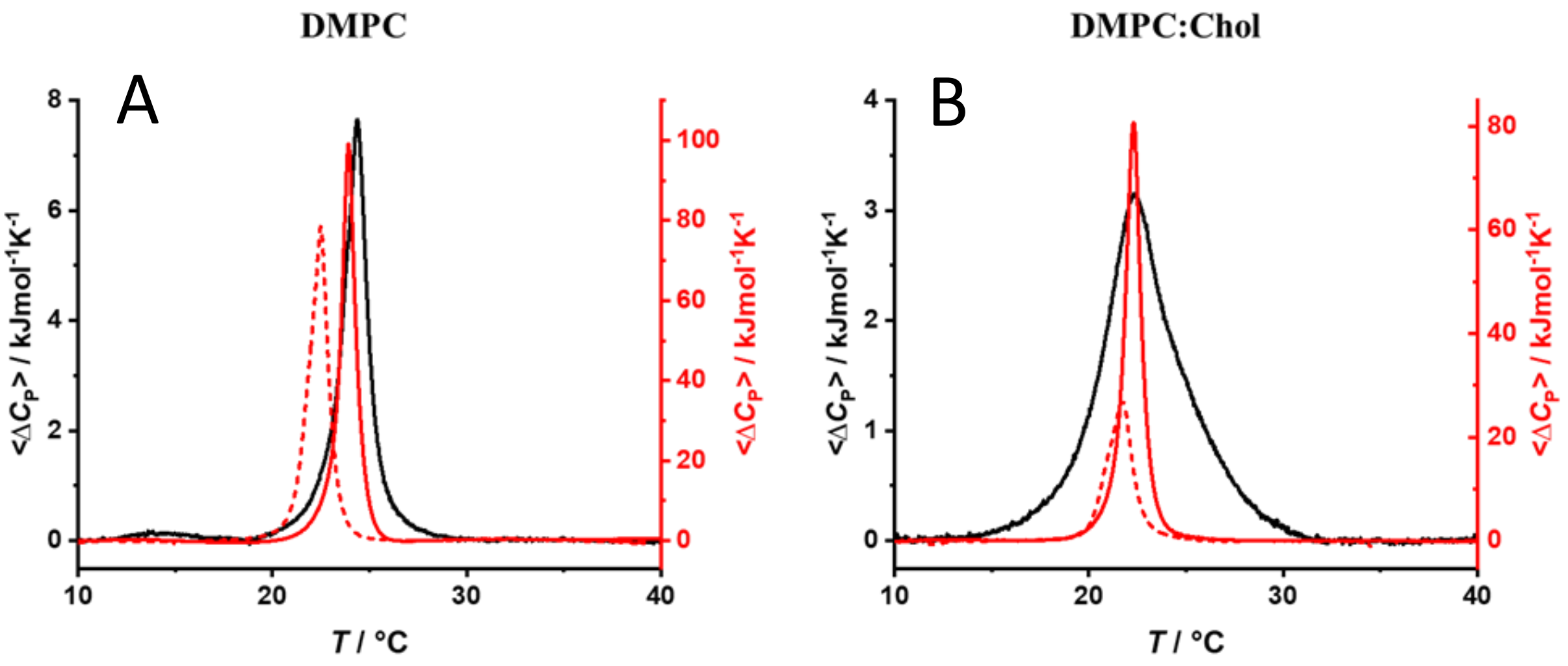

Figure S2. DSC curves of TF-liposomes (black curve) and μF-liposomes (red curves), before (continuous line) and after dialysis (dashed line), for DMPC (**panel A**) and DMPC:Chol (**panel B**). Note the two different y-axis used to better highlight differences between TF- and -μF-liposomes.

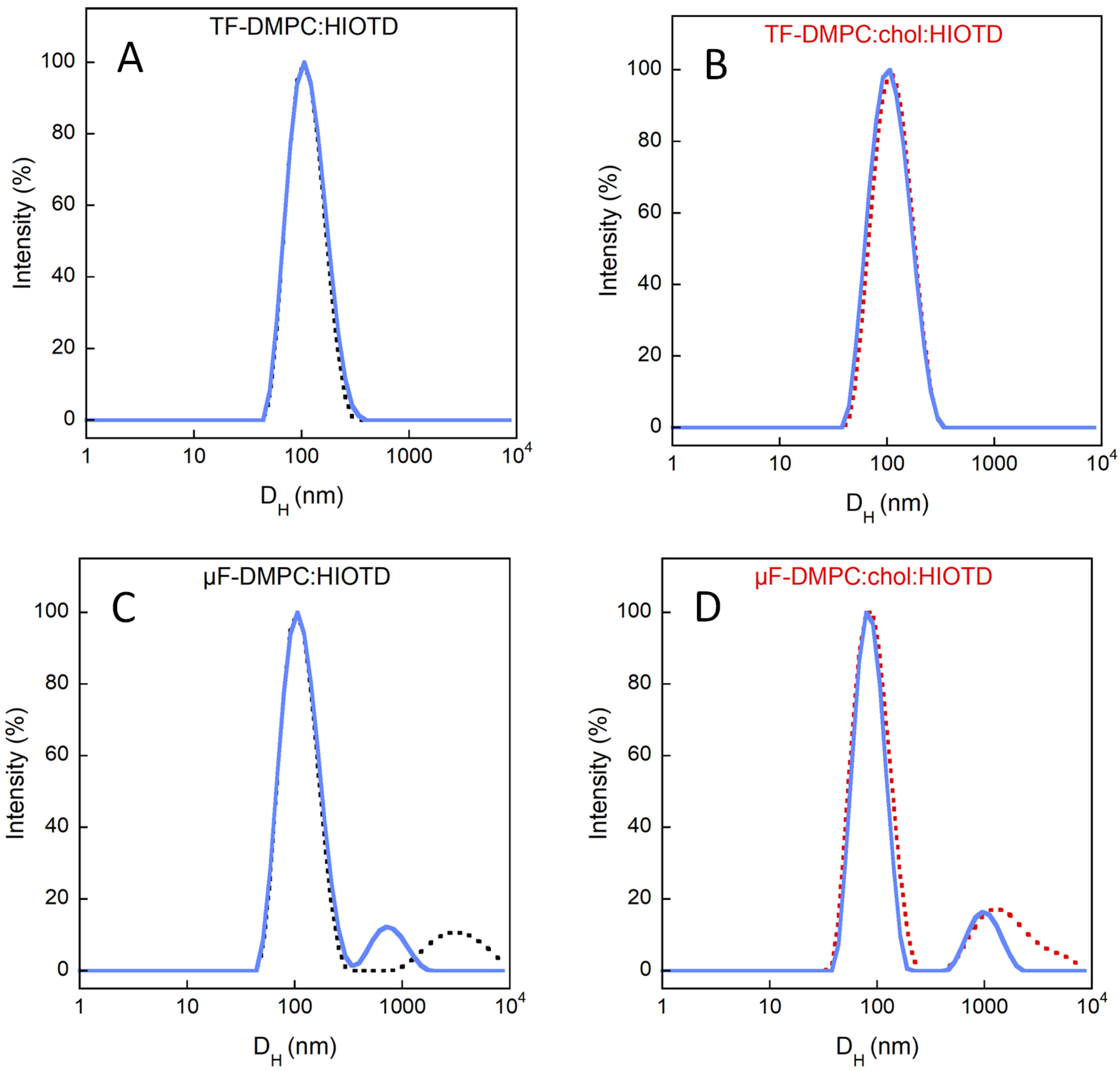

Figure S3. Intensity-weighted NNLS size distribution of liposomes after incubation with HIOTD: TF-DMPC:HIOTD (**panel A**), TF-DMPC:chol:HIOTD (**panel B**), both freshly prepared (black and red dotted profile, respectively); μF-DMPC:HIOTD (**panel C**), μF-DMPC:chol:HIOTD (**panel D**) immediately after dialysis (black and red dotted profile, respectively). The solid blue lines represent the same samples analyzed 20 days after preparation. HIOTD was incorporated into liposomes by incubation with a final lipid:HIOTD molar ratio of 8.3:1.7.

2. UV-Vis measurements of Cu(II) complexation with HIOTD

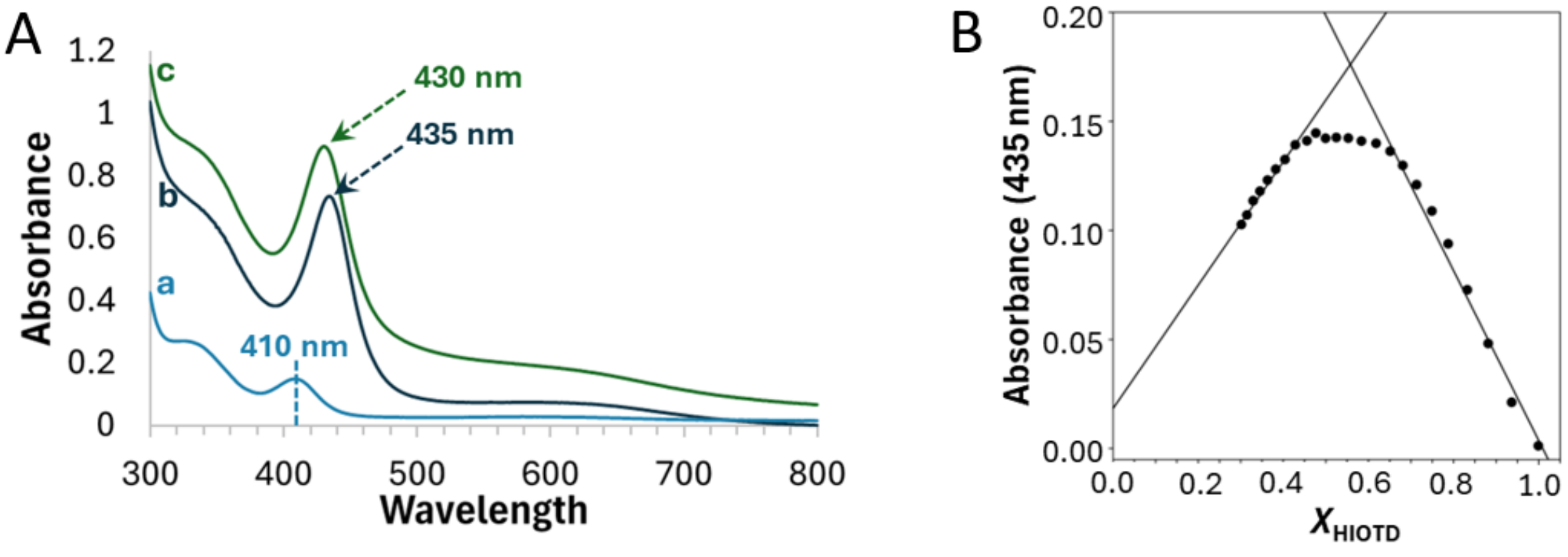

Figure S4. Panel A: UV-Vis spectra in ethyl acetate (**a**, **c**) and acetonitrile (**b**) of HIOTD-Cu(II) complex: **a.** HIOTD 7 mM in ethyl acetate stirred with an aliquot of $Cu(OAc)_2$ 20 mM in water (HIOTD / Cu(II) = 7:1, water / acetate = 4% (v/v)); **b.** HIOTD 7 mM, $Cu(OAc)_2$ 0.2 mM in acetonitrile; **c.** HIOTD 7 mM, $Cu(OAc)_2$ 0.2 mM in ethyl acetate. **Panel B**: Job plot for the determination of the HIOTD / Cu(II) stoichiometry of the complex in acetonitrile with TBAF 15 mM ionic strength buffer. Aliquots of a $Cu(OAc)_2$ 0.2 mM were added to HIOTD 0.2 mM to record each spectrum, so that the ion + ligand total concentration is kept constant throughout the experiment.

Because HIOTD is hydrophobic, Cu(II) complexation has been studied in organic solvents of different polarity, acetonitrile and ethyl acetate. $Cu(OAc)_2$ was used as the copper source, so that the acetate counterion serves as proton acceptor upon formation of the complex [1].

HIOTD-Cu(II) complexes exhibit a characteristic broad band at about 610 nm (Figure S4, panel A: Spectra of HIOTD 7 mM in acetonitrile or ethyl acetate with $Cu(OAc)_2$ 0.2-1.0 mM) associated with the d-d electronic transition of Cu(II), and a sharper and more intense band at 400-450 nm associated with the ligand-to-metal charge transfer transition (LMCT).

Spectra **b** and **c** in panel A of Fig. S4 were obtained, respectively, in acetonitrile and ethyl acetate at HIOTD 7 mM and $Cu(OAc)_2$ 0.2 mM. Samples preparation was as follows: $Cu(OAc)_2$ was dissolved in ACN or ethyl acetate to obtain 20 mM stock solutions. A clear solution was obtained in ACN, whereas a cloudy suspension resulted in ethyl acetate. However, the solid was finely and uniformly dispersed and the aliquots could be easily sampled. Spectra **b** and **c** show that changing the solvent from acetonitrile to less polar ethyl acetate causes a 5 nm blue shift of the LMCT band of the HIOTD-Cu(II) complex (from 435 to 430 nm).

Spectrum **a** was also obtained with HIOTD in ethyl acetate (7 mM, same as spectrum **c**). However, in this case $Cu(OAc)_2$ was dissolved in double distilled water, added to HIOTD and partitioned in the

organic phase to form the HIOTD-Cu(II) complex. A 30 minutes wait was necessary to allow settling of the aqueous droplets and to obtain reproducible spectra. Thereby, spectrum **a** reflects the formation of the HIOTD-Cu(II) complex with aqueous Cu(II) in an apolar environment. Remarkably, the maximum of the LMCT band is found at 410 nm, in keeping with one of the bands observed when the complex is formed in the liposomes (see main text).

The Job plot (Fig. S4, right) for the determination of the stoichiometry of the complex was obtained as follows in acetonitrile [2]. First, we checked that the 435 nm band follows the Lambert-Beer law as a function of $Cu(OAc)_2$ concentration, as required in the method of continuous variations. In a 1 cm optical path length quartz cuvette, 20-100 mL aliquots of a 5 mM $Cu(OAc)_2$ stock solution were added to 7 mM HIOTD and a spectrum was recorded after each addition. Cu(II) concentrations were 0.04-0.2 mM. The plot of A435 vs. [Cu(II)] is linear and allowed us to calculate e435 = 3807 M-1cm-1 (data not shown).

For the construction of the Job plot, tetrabutylammonium tetrafluoroborate (TBAF) was used to buffer the ionic strength. 0.2 mM solutions of $Cu(OAc)_2$ and of HIOTD were prepared in CH_3CN / TBAF 15 mM. Aliquots of Cu(II) solution were added to HIOTD in the cuvette and spectra were recorded at 25°C after each addition. With the last aliquot, taking dilution into account, Cu(II) was 0.14 mM and HIOTD was 0.06 mM. In the Job plot of absorbance vs. molar fraction X_{HIOTD} no sharp maximum is detected, so we approximated its position through the intersection of the two linear parts of the curve corresponding to defect and excess ligand, respectively. We thereby calculated $X_{HIOTD} \approx 0.55$, i.e a 1:1 ligand / metal stoichiometry. The curvature of the maximum of the Job plot suggests binding of intermediate strength.

3. UV-Vis blank experiments: HIOTD aggregates and HIOTD-free DMPC liposomes

Blank experiments were performed to highlight any spectral feature related to the interaction of non-liposomial HIOTD and HIOTD-free TF- and µF-liposomes. Detailed procedure are in the Experimental section in the main text.

HIOTD was suspended in PB (0.3 mmoles L^{-1}), then aliquots of $Cu(OAc)_2$ 20 mM stock solution in water were added and spectra were recorded against PB after each addition (Fig. S5 B). The presence of insoluble material (both HIOTD and Cu(II) salts) produces significant noise in the spectrum.

Aliquots of $Cu(OAc)_2$ 20 mM stock solution were added to 2 mM DMPC TF- and µF-liposomes and spectra were recorded after each addition (Fig. S5 C and D, respectively).

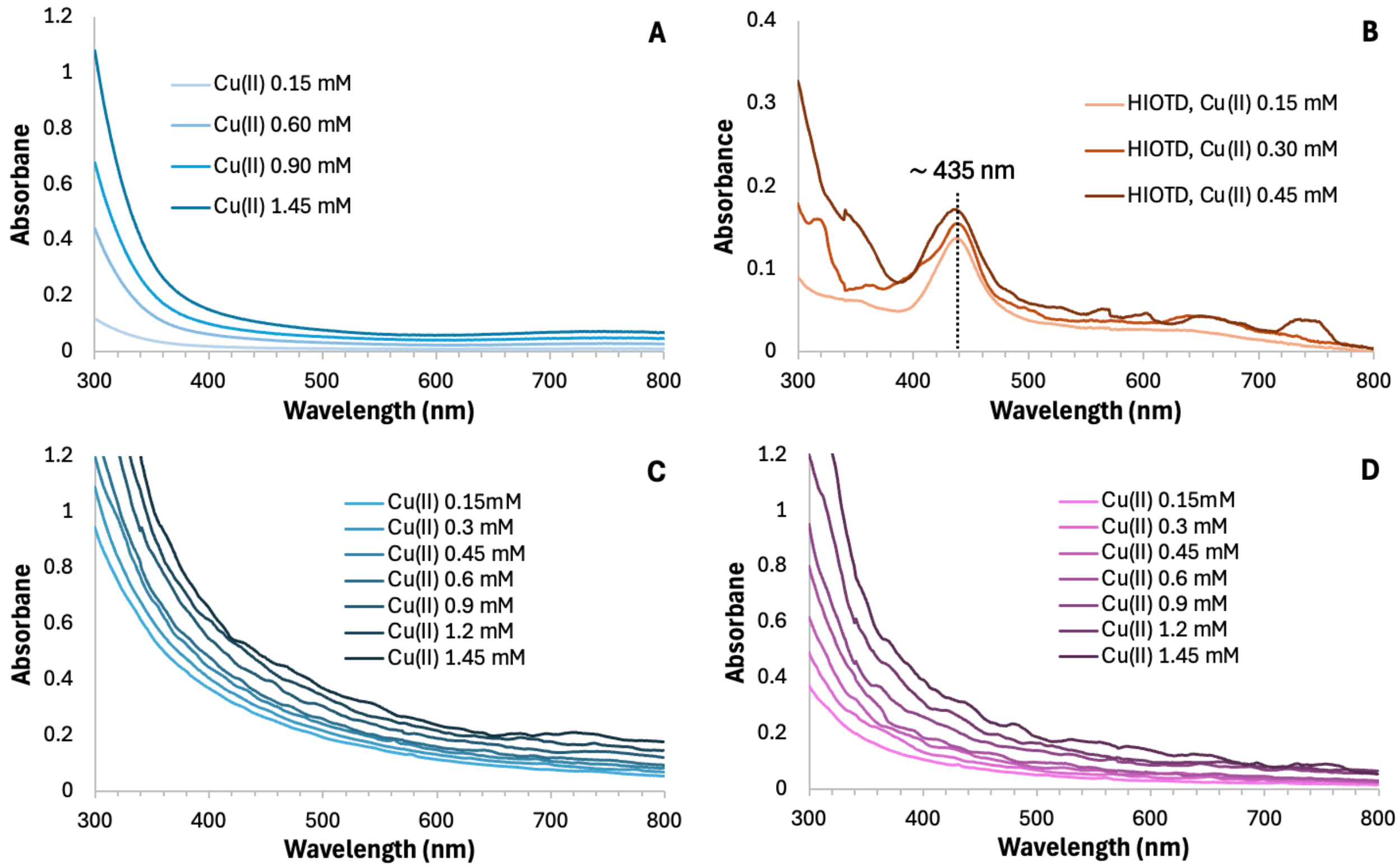

Figure S5. UV-Vis spectra recorded at increasing Cu(II) concentrations in PB. **Panel A**) $Cu(OAc)_2$ only; **panel B**) HIODT aggregates; **panel C**) TF-DMPC liposomes and **panel D**) µF-DMPC liposomes.

Figure S6 shows the intensity weighted size distribution of TF liposomes for an intermediate copper concentration (0.6 mM), compared to the one measured in the absence of copper.

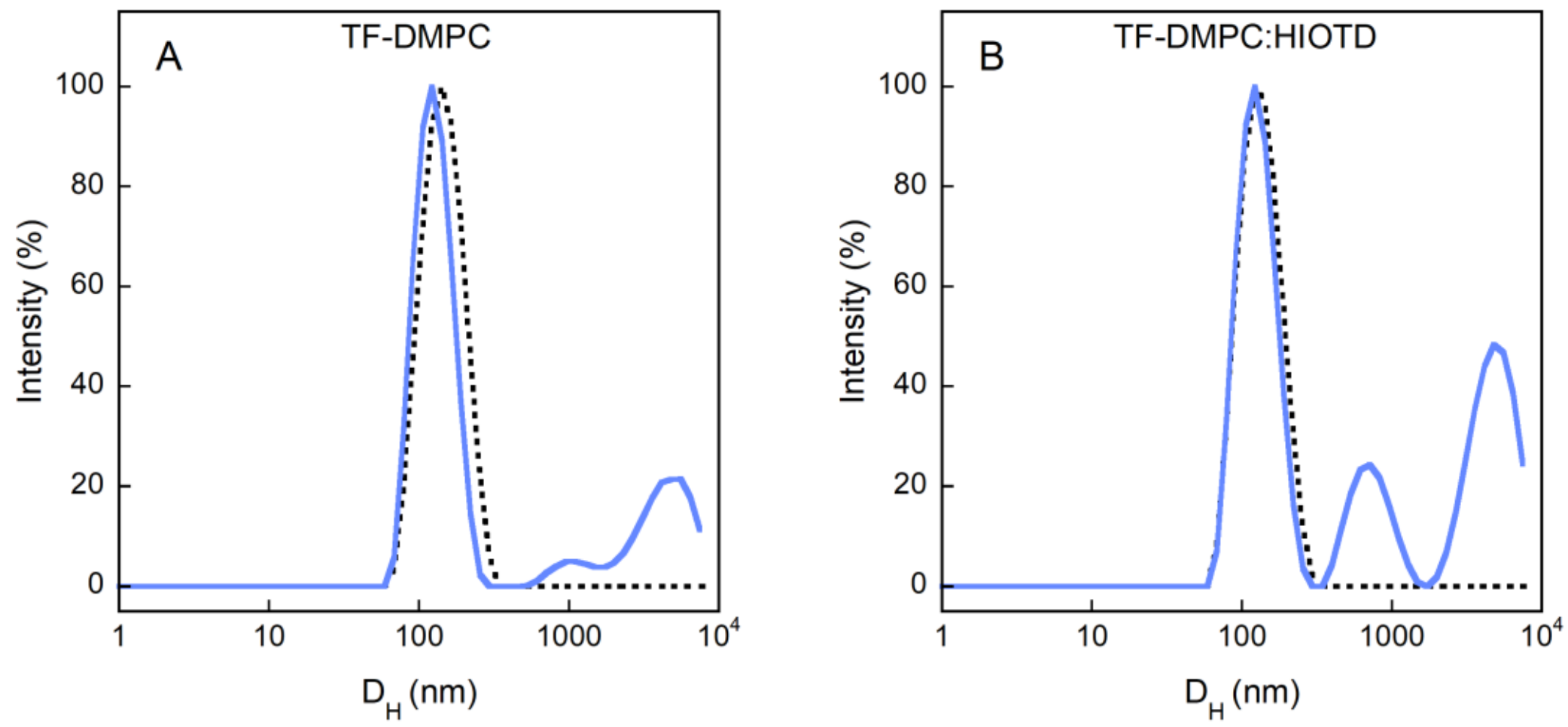

Figure S6. Intensity-weighted NNLS size distribution of TF-DMPC (**panel A**) and TF-DMPC:HIOTD liposomes (**panel B**). Liposomes in the absence of Cu (II) (black dotted profile) and in presence of Cu (II) 0.6 mM (solid blue profile).

References

[1] I. Antignano, S. Casciardi, F. D'Acunzo, A. Del Giudice, L. Gatti, P. Gentili, F. Mura, A. Ricci, G. Masci, Hybrid copper-polyelectrolyte nanoaggregates obtained with smart block copolymers based on 4-[(hydroxyimino)aldehyde]butyl methacrylate (HIABMA) in water and acetonitrile, Polymer 306 (2024) 127197. https://doi.org/10.1016/j.polymer.2024.127197.

[2] J.S. Renny, L.L. Tomasevich, E.H. Tallmadge, D.B. Collum, Method of Continuous Variations: Applications of Job Plots to the Study of Molecular Associations in Organometallic Chemistry, Angew Chem Int Ed 52 (2013) 11998–12013. https://doi.org/10.1002/anie.201304157.
